# Long non-coding RNA generated from *CDKN1A* gene by alternative polyadenylation regulates p21 expression during DNA damage response

**DOI:** 10.1093/nar/gkad899

**Published:** 2023-10-23

**Authors:** Michael R Murphy, Anthony Ramadei, Ahmet Doymaz, Sophia Varriano, Devorah M Natelson, Amy Yu, Sera Aktas, Marie Mazzeo, Michael Mazzeo, George Zakusilo, Frida E Kleiman

**Affiliations:** Chemistry Department, Hunter College, The City University of New York, New York, NY 10021, USA; Biology Program, The Graduate Center, The City University of New York, New York, NY 10016, USA; Chemistry Department, Hunter College, The City University of New York, New York, NY 10021, USA; Biology Program, The Graduate Center, The City University of New York, New York, NY 10016, USA; Chemistry Department, Hunter College, The City University of New York, New York, NY 10021, USA; Chemistry Department, Hunter College, The City University of New York, New York, NY 10021, USA; Biology Program, The Graduate Center, The City University of New York, New York, NY 10016, USA; Chemistry Department, Hunter College, The City University of New York, New York, NY 10021, USA; Biology Program, The Graduate Center, The City University of New York, New York, NY 10016, USA; Chemistry Department, Hunter College, The City University of New York, New York, NY 10021, USA; Chemistry Department, Hunter College, The City University of New York, New York, NY 10021, USA; Chemistry Department, Hunter College, The City University of New York, New York, NY 10021, USA; Chemistry Department, Hunter College, The City University of New York, New York, NY 10021, USA; Chemistry Department, Hunter College, The City University of New York, New York, NY 10021, USA; Chemistry Department, Hunter College, The City University of New York, New York, NY 10021, USA; Biology Program, The Graduate Center, The City University of New York, New York, NY 10016, USA

## Abstract

Alternative Polyadenylation (APA) is an emerging mechanism for dynamic changes in gene expression. Previously, we described widespread APA occurrence in introns during the DNA damage response (DDR). Here, we show that a DDR-activated APA event occurs in the first intron of CDKN1A, inducing an alternate last exon-containing lncRNA. We named this lncRNA SPUD (Selective Polyadenylation Upon DNA Damage). SPUD localizes to polysomes in the cytoplasm and is detectable as multiple isoforms in available high-throughput studies. SPUD has low abundance compared to the CDKN1A full-length isoform under non-stress conditions, and SPUD is induced in cancer and normal cells under a variety of DNA damaging conditions in part through p53. The RNA binding protein HuR binds to and promotes the stability of SPUD precursor RNA. SPUD induction increases p21 protein, but not mRNA levels, affecting p21 functions in cell-cycle, CDK2 expression and cell growth. Like CDKN1A full-length isoform, SPUD can bind two competitive p21 translational regulators, the inhibitor calreticulin and the activator CUGBP1; SPUD alters their association with CDKN1A full-length in a DDR-dependent manner, promoting CDKN1A translation. Together, these results show a new regulatory mechanism by which a lncRNA controls p21 expression post-transcriptionally, highlighting lncRNA relevance in DDR progression and cell-cycle.

## Graphical Abstract

**Graphical Abstract:**
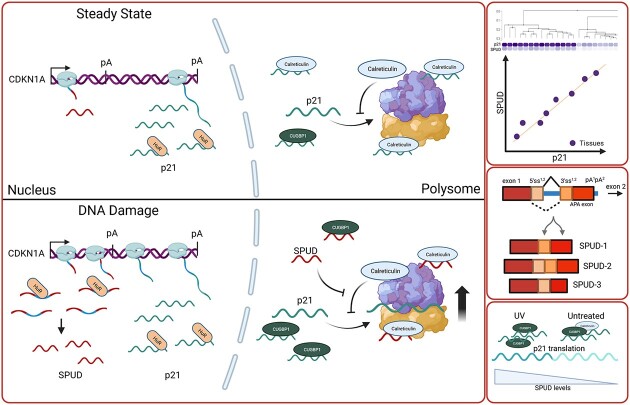

## Introduction

The annotated human genome contains almost 200 000 transcripts from nearly 60 000 genes (1,2). Many of these additional transcripts are expressed from protein-coding genes as a product of alternative RNA processing, including splicing and polyadenylation (3,4). Alternative isoforms from the same protein-coding gene have the potential to generate distinct transcripts and alter cellular mRNA composition, while utilizing the same transcription and RNA processing machineries (5). These distinct transcripts may take the form of long non-coding RNAs (lncRNAs), which are characterized by poor conservation, low abundance (relative to mRNAs), and their induction in specific settings, such as certain tissues or in response to stress (6–8). The transient nature of lncRNA induction facilitates the fine-tuning of different cellular responses, such as the type and length of DNA damage response (DDR) (9,10).

Several lncRNAs have been described in close proximity to *CDKN1A* (11), the gene that codes for p21, that function either *in cis* affecting *CDKN1A* transcription or *in trans* by binding to other DDR factors (12–15). The cyclin-dependent kinase (CDK) inhibitor p21 has a variety of functions in cell-cycle regulation and DDR, playing a role in cell fate decision between senescence and apoptosis after DNA damage (reviewed in (16). In fact, p21 is a dose-sensitive ‘goldilocks protein’ which can be oncogenic at levels that are either too low or too high, playing a key factor in chemotherapy effectiveness by inducing the cells to either enter senescence or maintain a proliferative state (17,18). Thus, *CDKN1A*, and perhaps other cell-cycle genes, exist as hubs for non-coding transcription to regulate cellular functions, such as DDR and cell-cycle progression, in addition to canonical protein production. *CDKN1A* expression is tightly regulated transcriptionally by p53. A delay in *CDKN1A* mRNA induction and p21 expression has been described for various stresses, such as UVC and during S-phase block with hydroxyurea (HU), despite comparable levels of RNA polymerase II and p53 at *CDKN1A* promoter to other stresses (19,20). The delay in *CDKN1A* full-length mRNA induction is in part due to a block in transcription elongation somewhere in intron 1 (21–24). Whether the truncated CDKN1A transcript is processed or degraded has not been addressed.

Alternative polyadenylation (APA) is an effective regulator of cellular homeostasis (reviewed in (25–27). Approximately 20% of human genes have an intronic polyadenylation signal (PAS) with many tied to an alternate splicing event (alternate last exon; ALE, (28). A considerable number of promoter-proximal APA events in introns (intron-APA) occur during stress and in cancer cells, leading to truncated transcripts and alternative C-termini protein products (29–31). Interestingly, intron-APA can also generate non-coding RNAs that regulate its own protein-coding mRNA in response to UV damage (5). Increase in intron-APA events mediated by U1 snRNA have been described (32,33). Our lab previously described an UV-induced increase in intron-APA events, which are biased to genes with functions in DDR and cancer, including *CDKN1A* (34). It is not known whether these transcripts represent functional products of DDR or passive units generated during the suppression of transcription/processing of canonical mRNA; however, the high representation of intron-APA transcripts in RNAseq datasets and the presence of strong PAS sites in promoter-proximal introns suggests that these transcripts are indeed functional (35).

Here, we provide insights into the truncated *CDKN1A* transcript generated by intron-APA after UV-treatment, which we named *S*elective *P*olyadenylation *U*pon DNA *D*amage (SPUD, 34). Our results indicate that SPUD is an ALE-containing lncRNA regulated by p53, which localizes to polysomes and is detectable in different datasets. Under a variety of damaging conditions, SPUD has comparable abundance relative to *CDKN1A* full-length isoform, SPUD is detectable in normal tissues and cancer cells. SPUD is regulated post-transcriptionally by HuR, SPUD can also functionally interact with the p21 translational regulators calreticulin (CRT) and CUGBP1, altering p21 expression. Consistent with SPUD regulating p21, manipulating SPUD levels alters cell cycle and cell growth. Together, our study reveals a new mechanism by which lncRNA SPUD regulates p21 expression at the translational level, highlighting SPUD relevance in cellular functions such as DDR progression and cell-cycle.

## Materials and methods

### Cell lines, plasmids and treatments

HCT116, HCT116 p21−/−, HCT116 p53−/−, BJ, MCF7 and MDA-MB-231 cells were grown as previously described (34). HEKa cells were from ATCC. HCT116 p21−/− cells were generously provided by Dr. Bert Vogelstein (Johns Hopkins University, Baltimore). APA isoforms were cloned into mammalian p3xFLAG-CMV10 and bacterial pET-42a(+) vectors. Mammalian vectors were treated with endotoxin removal component of Qiagen kit before transfections. UV treatment (20 Jm^2^) were as in (34). 10 μM etoposide (MilliporeSigma) were added directly to media 16 h before harvesting. CUGBP1 and CRT expressing constructs were generously provided by Dr Wilusz (University of Colorado) and Dr. Michalak (University of Alberta), respectively.

### Plasmid mutagenesis

CDKN1A intron 1 APA 3′ splice site was deleted using Q5 Site-Directed Mutagenesis Kit (New England Biolabs) as per the manufacturer's instructions using forward primer (5′-TCCCCACCCCAAAATGACGCGCAGCC-3′) and reverse primer (5′-GGGGGAGAATGGGAGGGG-3′). Stem loop mutant was created with the following primers (5′-CGCGCGTGATTTCCTGGGCGGGAATAGCAC-3′ and (5′-GTGCTATTCCCGCCCAGGAAATC ACGCGCG-3′). Plasmids were sequenced to confirm the presence of the mutation.

### RNA extraction and qRT-PCR assays

The qRT-qPCR data was assured by following the MIQE guidelines as follows (36). Total RNA was extracted using TRIzol (Invitrogen) according to the manufacturer's instructions as described (34) from all the cells used in this study that were mock- or treated (UV, Actinomycin D, etoposide, cycloheximide). Reverse transcription (RT) was performed with M-MLV reverse transcriptase (Promega) in a programmable thermal controller (MJ Research). RNA was quantified using nanodrop and 100 ng of *A*_260_/*A*_280_ >1.95 of freshly prepared or one-time thawed RNA was used for reverse transcription. Freshly prepared or one-time thawed cDNA was diluted 1:10 prior to PCR amplification and then subjected to real time PCR in-house in a in StepOnePlus (ThermoFisher) Real-Time PCR Detection System using PowerUP SYBR Green PCR Master Mix (Applied Biosystems) as described previously (34). Non reverse transcriptase-containing RNA samples were used as controls for gDNA or plasmid contamination during real time PCR. All reactions were carried out as technical triplicates of biological triplicates and data was deemed viable for a standard deviation of <0.6 between technical replicates. Sequences of primers used are listed in Supplementary Figure S5. Individual amplification curves were determined for each amplicon. Data were normalized to reference genes (Ubiquitin C (UBC) or β-Actin) by 2-ΔΔCt method (37).

### Knockdown expression

siRNA specific for human HuR, a custom-made siRNA for SPUD and a control siRNA were obtained from Dharmacon RNA technologies. Knockdowns were done according to manufacturer's protocol (Invitrogen) and tested by qRT-PCR and western blot. SPUD knockdowns we tested by qRT-PCR.

### RNA stability assay

RNA was analyzed by qRT-PCR from cells treated with UV irradiation (20 Jm^2^), allowed to recover for 2 h and then treated with 2 μg/ml actinomycin D into the existing media as described in (38).

### Protein extraction, western blot analysis and antibodies

Nuclear and cytoplasmic protein extracts were prepared as described in (39). Samples were analyzed by immunoblotting with mAbs targeted against p21 (N-20; Santa Cruz), PARP (9542; Cell Signalling), HuR (3A2; Santa Cruz), FLAG (4GFR; GeneTex), GAPDH (G9; Cell Signalling), Lamin A (H-102; Santa Cruz), CRT (F-4; Santa Cruz), CUGBP1 (B-1; Santa Cruz)

### 3′ and 5′ Rapid amplification of cDNA ends (3′RACE and 5′RACE)

Nuclear RNA was analyzed with the 3′ RACE System for Rapid Amplification of cDNA ends (ThermoFisher) as per the manufacturer's protocol using oligo(dT)-adaptor primer (5′-GGCCACGCGTCGACTAGTACTTTTTTTTTTTTTTTTT-3′). PCR amplification was done using CDKN1A Exon 1 specific primer (5′-ATGCGTGTTCGCG GGTGT-3′) located 400 bp upstream of poly(A) site and adaptor (5′-GGCCACGCGTCGA CTAGTAC 3′). For nested PCR, a second CDKN1A specific forward primer in SPUD alternative exon was used (5′-AGCCGGAGTGGAAGCAGA-3′) and the same adaptor. Cytoplasmic RNA from HCT116 cells was analyzed for 5′RACE using a forward primer located in the 5′ UTR of the CDKN1A gene (5′-GAGGTGTGAGCAGCTGCCGAAG-3′) and a reverse primer in either exon 2 or in SPUD ALE.

### Protein translation assays

FLAG-tagged SPUD *in vitro* expression was done with Transcend Non-Radioactive Translation Detection System (Promega) as per the manufacturer's instructions. Expression was analyzed by chemiluminescence following a reaction of Lysine-biotin bound streptavidin-HRP to the substrate. *In vivo* expression was analyzed by Western blot with anti-FLAG. p21 was generated by *in vitro* translation assays in rabbit reticulocyte lysate (ThermoFisher) using cDNA transcribed by T7 polymerase from human *CDKN1A* gene expression plasmid (Sino Biological US). Samples were generated as in (40). Six microliters of p21 lysate were incubated with or without addition of equal molarity of GST-CRT and/or GST-CUGBP1. Additionally, SPUD or ‘unspliced SPUD’ were added to the reaction. Samples were analyzed by Western blot.

### RNA immunoprecipitation (RIP)

The IP of nuclear RNA-protein complexes was performed as described (41). After treatments, cells were cross-linked and nuclear extracts were prepared. Extracts were treated with DNase (Ambion), and the resulting material was IP’ed with monoclonal antibodies against HuR (3A2; Santa Cruz), CRT (F-4; Santa Cruz), CUGBP1 (B-1; Santa Cruz) or control rabbit IgG (Sigma). Protein-RNA complexes were treated with proteinase K and reversal of cross-linking. The RNA was extracted from the IPs with phenol-chloroform and analyzed by RT-qPCR assays.

### RNA pull-down (RPD)

T7-driven pET-42a(+) construct containing SPUD transcript was used for *in vitro* transcription using RNA Biotin Labelling Mix (Sigma) and T7 polymerase (Promega) following manufacturer's instructions. Biotinylated RNA was incubated with either nuclear extracts from HCT116 cells or recombinant His-HuR, GST-CRT, or GST-CUGBP1 and then pulled-down with magnetic streptavidin beads (ThermoFisher) followed by immunoblotting analysis, as described (41).

### Flow cytometry

Treated HCT116 cells were fixed and treated with staining mixture (0.1% Triton X-100, 2 mg RNAse A, 20 μg/ml propidium iodide). Samples were analyzed on FACSCalibur Flow Cytometer (BD Biosciences).

### RNA-seq/Ribo-Seq analysis

Publicly available RNA-seq and Ribo-seq dataset was downloaded from gene expression omnibus (NCBI) under accession number GSE99745 (42) and GSE198792. Downloaded fastq files were aligned to hg38 human genome using STAR and visualized using IGV (Broad Institute). Proportions of splice junctions used per sample were analyzed using IGV’s integrated sashimi plot function and taken as a percentage of the junction reads detected for each isoform.

### Statistical Methods

Statistical significance between experimental groups was ascertained using GraphPad Prism 9.0. Results from qRT-PCR, Western blotting and flow cytometry data were performed in three or more independent biological samples analyzed by triplicate, presented as mean ± SEM. Experiments with two groups were analyzed using two-tailed unpaired Student's t-test. In the presented data, one (*), two (**), three (***) and four (****) corresponded to *P* < 0.01, *P* < 0.001, *P* < 0.0001 and *P* < 0.00001, respectively.

## Results

### SPUD is a UV-inducible intragenic transcript generated by an intron-APA event in *CDKN1A* gene

**Figure 1.**
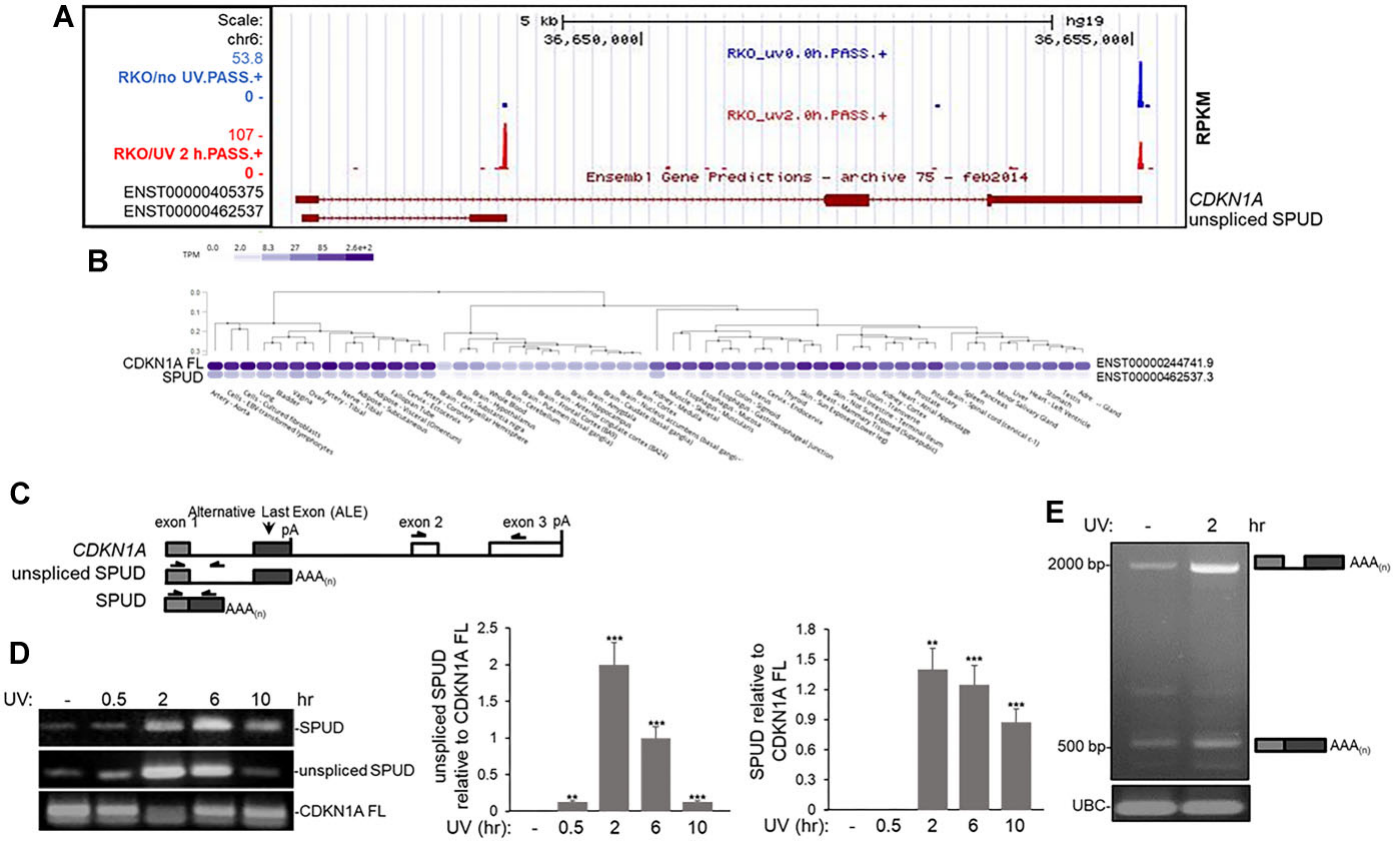
UV-mediated APA event in *CDKN1A* intron 1 generates a processed transcript with an ALE. **(A)** Screenshot from the UCSC genome browser indicates an annotated transcript terminating at intron 1 (maroon diagram at the bottom). This intron-APA uses the same poly(A) signal in intron1 that the transcript detected via 3′ READS technique in samples from HCT116 cells treated with UV and allowed to recover for 2 h (20 J/m²). Detection was scored in Reads Per Million (RPM), calculated as the number of poly(A) site supporting (PASS) reads of hat site in a million unique PASS reads per sample. Blue and red bars indicate 3′READS CpA detection before and after UV treatment, respectively. Maroon boxes are exons and serrated lines are introns. Longer transcript on the top corresponds to *CDKN1A* full-length mRNA. **(B)** Detection of SPUD in a variety of different tissues. Shown is a graphic from GTEx portal on different *CDKN1A* isoforms expression. Blue ovals indicate the transcripts per million (TPM) across all individuals sequenced for each respective tissue. ENST0000244741.9 represents full-length isoform expression. SPUD (ENST00000462537.3) is represented by a single transcript according to GTEx. Dendrograms indicate tissues that cluster according to *CDKN1A* isoforms expression. **(C)** Schematic of PCR strategies to detect *CDKN1A* full-length mRNA (top), unspliced intron-APA transcript (unspliced SPUD) and spliced intron-APA transcript (SPUD) for semi-quantitative and quantitative PCR. For ‘unspliced SPUD’: forward primer was in exon 1 and reverse primer was downstream of exon 1 within intron 1, for SPUD: forward primer was in exon 1 and reverse primer was in the Alternative Last Exon (ALE), and for full-length CDKN1A: forward primer was in exon 2 and reverse primer was in exon 3. Isoform distinction is based on molecular weight. The identity of the bands was confirmed by sequencing. Isoform distinction is based on molecular weight. **(D)** Semi-quantitative analysis indicates that ‘unspliced SPUD’ and SPUD levels transiently increase after DNA damage without concomitant significant increase in full-length *CDKN1A* mRNA. Left: HCT116 cells were treated with UV (20 J/m^2^) and allowed to recover for indicated time points followed by RT-PCR using purified RNA and primer sets described in (C). cDNA was prepared using oligo(dT) primers. Right: ratio of ‘unspliced SPUD’ or SPUD to *CDKN1A* full-length mRNA. Values were normalized to non-treated cells. A representative gel from 3 independent biological samples analyzed by triplicate is shown. Errors represent SD (*n* = 3). **P < 0.001 and ***P < 0.0001. **(E)** Spliced transcripts generated from APA-ALE are detectable after UV treatment (20 J/m^2^, 2 h recovery) in samples from HCT116 cells by RT-PCR using SPUD primer set as in (C). Schematic next to gel indicates the SPUD isoform detected. Ubiquitin C (UBC) primers were used as loading control. A representative gel from 3 independent biological samples analyzed by triplicate is shown.

Previously, we described a strong activation of intron-APA sites after UV-treatment in colon carcinoma RKO cells, resulting in widespread expression of truncated transcripts, including from *CDKN1A* gene (34). As the coding sequence for p21 begins downstream in exon 2, the UV-induced APA event generated an entirely distinct, truncated transcript that we named *S*elective *P*olyadenylation *U*pon DNA *D*amage (SPUD). Interestingly, a two-exon expressed sequence tag (EST; ENST00000462537) terminating adjacent to a PAS was identified by 3′ region and extraction deep sequencing (3′READS, (43) and was present in UCSC Browser (44,45, Figure 1A, Supplementary Figure S1A, 34). SPUD (ENST00000462537) is expressed across multiple human tissues in a pattern similar to *CDKN1A* full-length according to Genotype-Tissue Expression (GTEx, Figure 1B), highlighting the existence of an uncharacterized *CDKN1A* transcript isoform detected in cell culture models (34) and in different tissues under non-stress conditions.

Inspection of ENST00000462537 indicated the presence of a canonical 3′ splice site (3′ss, CAG/GG) and the presence of two tandem PAS; AUUAAA and AAUAAA located ∼57 nt and ∼28 nt upstream, respectively, of the EST 3′ end (Supplementary Figure S1A, (28,46). SPUD isoforms; unspliced and spliced, were detected by RT-PCR in samples from HCT116 cells treated with UV irradiation (20 J/m^2^) and allowed to recover for the indicated time points (Figure 1C). A forward primer targeting exon 1 and a reverse primer either in the intron or in ALE was used in RT-PCR (Figure 1C). The levels of ‘unspliced SPUD’ increased from 0 to 6 h, reaching basal level detection by 10 h (Figure 1D), similar to the time course previously described for cleavage/polyadenylation inhibition during DDR (40). Similarly, the spliced isoform, SPUD, was strongly induced 2 h after UV treatment but still prevalent 10 h post-UV (Figure 1D). Using ALE reverse primer, RT-PCR analysis showed a smaller band (∼500 bp), which corresponded to SPUD (predicted spliced two-exon transcript; ENST00000462537), and a larger band (∼2 kb) that corresponded to ‘unspliced SPUD’ or precursor (Figure 1E). A third band was also detected (∼750 bp) that did not respond to UV treatment. The use of EST 3′ splice site (3′ss, CAG/GG) in the ∼500 bp band was confirmed by Sanger sequencing (data not shown).

**Figure 2.**
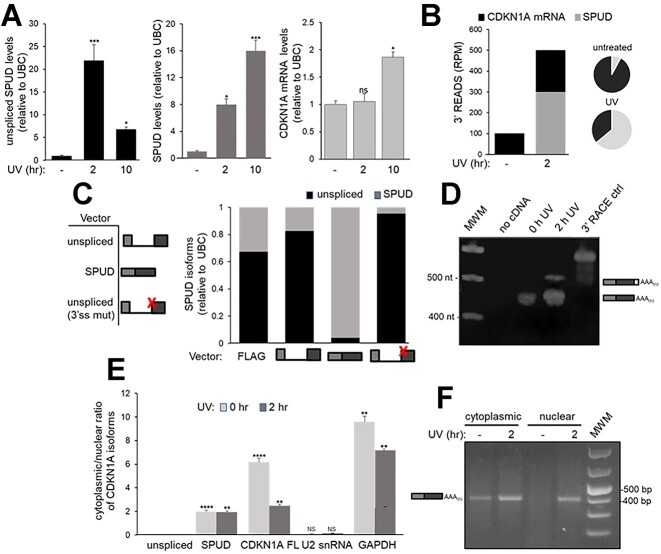
SPUD isoforms with multiple PAS and splicing elements and *CDKN1A* full-length expression levels show different patterns after UV-treatment. **(A)** While UV-transiently induces ‘unspliced SPUD’, SPUD can be detected longer in DDR progression. HCT116 cells were treated with UV (20 J/m²) and then recovered at times indicated followed by qRT-PCR using primers described in Supplementary Figure S5. cDNA was synthesized using oligo(dT) primers. The levels of the isoforms observed for each condition were normalized to the levels observed in non-treated cells. Three independent biological samples analyzed by triplicate is shown, SD (*n* = 3). *P < 0.01 and ***P < 0.0001. **(B)** Quantification of SPUD *versus CDKN1A* full-length transcript during DDR. Reads per million (RPM) values were determined as described in Figure 1A (34) for usage of intronic and 3′UTR PAS in *CDKN1A*. Left panel: values before and after UV; right panel: pie charts showing proportional abundances calculated via 3′READS. **(C)** The predicted 3′ss is responsible for the detection of SPUD. Amplified cDNAs from UV-treated HCT116 cells were used to clone ‘unspliced SPUD’ and SPUD into FLAG-tagged mammalian expression vectors. For ‘unspliced SPUD’: forward primer was in exon 1 and reverse primer was downstream of exon 1 within intron 1, for SPUD: forward primer was in exon 1 and reverse primer was in the Alternative Last Exon (ALE), and for full-length CDKN1A: forward primer was in exon 2 and reverse primer was in exon 3. ‘Unspliced SPUD’ vector was mutated to delete the CAGGG sequence shown in Figure S1A. Left: schematic of expression constructs; red ‘x’ indicating the vector with mutated 3′ss. Right: relative abundance of unspliced and SPUD in samples of each transfection condition was determined by qRT-PCR. cDNA was synthesized using oligo(dT) primers. Data shown is from 3 independent biological samples analyzed by triplicate (*n* = 3). **(D)** Both intronic PAS in *CDKN1A* are used after UV treatment. Samples from UV-treated HCT116 cells were analyzed for *CDKN1A* intronic polyadenylation by 3′RACE using a forward primer within exon 1 and a reverse oligo(dT)-tagged adapter primer. RNA was loaded as control for genomic DNA contamination. 3′RACE control cDNA provided by the kit was included. A representative gel from three independent assays from three biological samples is shown (*n* = 3). **(E)** While ‘unspliced SPUD’ localizes in the nucleus, SPUD localizes to the cytoplasm, and their distribution are unaffected by UV treatment. Nuclear and cytoplasmic RNA extraction was performed on samples from cells treated as in (A). qRT-PCR was performed as in (A). Cytoplasmic/nuclear ratio of 2^−ΔΔCT^ of equal RNA input. U2 snRNA and GAPDH were used as fractionation controls. Data shown is from 3 independent biological samples analyzed by triplicate (*n* = 3). **P < 0.001 and ****P < 0.00001. **(F)** Spliced and polyadenylated SPUD is detectable in the cytoplasm after UV treatment. RNA extraction from cellular fractions was done as in (E) and 3′RACE analysis was done as in (C), except for an additional nested PCR using a downstream forward primer also within exon 1 for the second PCR. Samples were analyzed on an agarose gel and detected by ethidium bromide staining. A schematic of the product detected is shown adjacent. A representative gel from 3 independent biological samples analyzed by triplicate is shown. Molecular weight standard (MWS) is also included.

While a strong induction in unspliced (∼20×) and spliced (∼8×) SPUD was observed 2 h after UV treatment by quantitative analysis, ‘unspliced SPUD’ approached basal levels by 10 h (Figure 2A, Supplementary Figure S1B, C). SPUD (spliced) decrease was delayed, reaching basal levels 24 h after UV treatment (Supplementary Figure S1D), To further establish approximate abundances of SPUD and full-length *CDKN1A* during DDR progression, we analyzed our prior 3′READS dataset (34) and determined that full-length encompassed >92% of the reads for *CDKN1A* isoforms in non-stress conditions (Figure 2B, Supplementary Figure S1E). However, 2 h after UV, SPUD increased by 31.7-fold accounting for 64% of the *CDKN1A* reads. Levels analyzed by qRT-PCR also suggested a narrowing of the difference between SPUD and full-length after UV. The discrepancy in the abundances detected by 3′READS and qRT-PCR is likely due to 3′READS detecting both unspliced and spliced induction simultaneously. Taken together, this data describes for the first time an intron-APA event in the *CDKN1A* gene following DNA damage that generates a transcript with an ALE.

### SPUD is detected in the cytoplasm after undergoing splicing and polyadenylation

To determine 3′ss functionality, mammalian expression vectors were constructed for either the ‘unspliced SPUD’ sequence, SPUD, or the unspliced sequence with a 5 nt deletion at the CAG/GG splice site (Figure 2C). Quantitative analysis showed an increase in SPUD levels was observed in samples from cells expressing ‘unspliced SPUD’ construct (Supplementary Figure S1F) with SPUD induction similar to that observed after UV (Figure 2A), indicating that the exogenously expressed transcript was spliced. However, expression of a vector with a mutated 3′ss abolished SPUD precursor splicing (Figures 2C and Supplementary Figure S1F) with SPUD levels similar to those observed in cells transfected with empty vector, confirming the role of the intronic 3′ss in *CDKN1A* to generate the ALE in SPUD. This was confirmed by semi-quantitative RT-PCR using a forward primer in the vector and ALE reverse primer (Supplementary Figure S1G). 3′RACE of endogenously activated SPUD after UV treatment showed two bands indicative of the usage of the two tandem PAS available ∼29 nt apart (Figure 2D).

As splicing and polyadenylation have been previously described to stimulate nuclear export (47), we next tested SPUD localization. ‘Unspliced SPUD’ was exclusively nuclear, as expected for unspliced RNAs (48). SPUD was enriched in the cytoplasm but at lower levels than the full-length mRNA (Figure 2E). 3′RACE with nested PCR also showed that SPUD was predominantly cytoplasmic and was induced after UV treatment in both cytoplasmic and nuclear fractions (Figure 2F). Together, these results indicate that SPUD is a cytoplasmic RNA.

### RNA binding protein (RBP) HuR regulates SPUD levels

**Figure 3.**
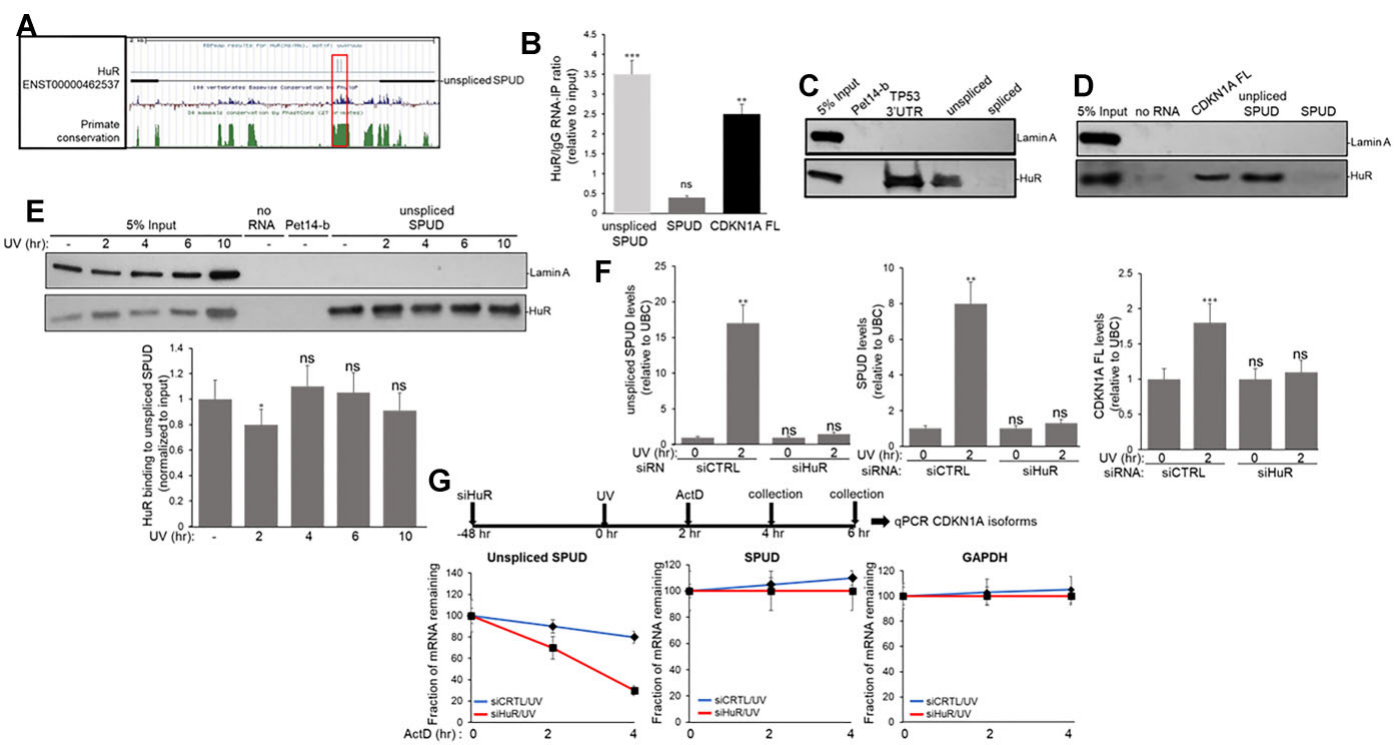
HuR interacts and regulates the stability of ‘unspliced SPUD’ independently of DNA damage. **(A)** RBPmap motif analysis highlighting the two potential HuR binding sites corresponding to the region of conservation detected in UCSC genome browser with multi-alignment of 100 vertebrate species showing regions of conservation along *CDKN1A* intron 1. Positive conservation as green peaks indicate region of conservation overlapping predicted HuR binding site. **(B)** HuR binds to unspliced but not SPUD isoform. HCT116 cells were treated with formaldehyde to generate protein-RNA cross-links. The samples were incubated with either anti-HuR or IgG antibodies. The endogenous nuclear RNA IP’ed with the antibodies was quantified by qRT-PCR using primers specific for each *CDKN1A* isomers described in Figure 1C. The qRT-PCR values were calculated from three independent biological samples analyzed by triplicate (*n* = 3). The input indicates samples before RIP. **P < 0.001 and ***P < 0.0001. (**C, D**) Unspliced but not spliced SPUD interacts with HuR independently of UV treatment. RNA pull-down assays were performed using *in vitro* transcribed biotinylated RNA from the *CDKN1A* APA isoforms mixed with NEs from HCT116 cells. Equivalent amounts of the eluate were resolved by SDS-PAGE and proteins were detected by immunoblotting using antibodies against Lamin A and HuR. *In vitro* transcribed biotinylated Pet14-b RNA was used as nonspecific sequence (no HuR sequence is present in this fragment), while RNA from TP53 and *CDKN1A* 3′UTRs were used as positive control for binding (96). Lamin A was used as negative control for binding. A representative gel from 3 independent biological samples analyzed by triplicate is shown. Five percent of the NEs used in the pull-down reactions is shown as input. **(E)** HuR binding to ‘unspliced SPUD’ is independent of UV treatment. Top: NEs from HCT116 cells exposed to UV (20 J/m²), allowed to recover for the indicated times were incubated with biotinylated SPUD and proteins were detected as in (B). Bottom: quantification of RNA pull-down assays represented in the top. The levels of HuR pulled down in each condition were normalized to the levels observed in each input. A representative pull-down reaction from 3 independent biological samples analyzed by triplicate is shown. Five percent of the NEs used in the pull-down reactions is shown as input. *P < 0.01 **(F)** siRNA-mediated knockdown of HuR prevents UV-induced activation of *CDKN1A* isoforms. RNA levels of SPUD isoforms and full-length *CDKN1A* mRNA were analyzed by qRT-PCR in samples from cells treated with HuR/control siRNA for 48 h and UV irradiation (20 J/m², 2 h recovery). RNA abundances were normalized to UBC. Data shown is from three independent biological samples analyzed by triplicate, SD (*n* = 3). **P < 0.001 and ***P < 0.0001 **(G)** HuR regulates abundance of *CDKN1A* APA isoforms in transcription-independent manner. ‘Unspliced SPUD’ stability is reduced upon HuR depletion. HCT116 cells treated with HuR/control siRNAs and UV irradiation as in (F). Cells were also incubated with actinomycin D for the indicated times after UV treatment followed by qRT-PCR of SPUD isoforms. mRNA decay rates for SPUD isoforms and GAPDH, a non-HuR target transcript, were determined by qRT-PCR at different time points following HuR/control siRNA-, UV- and Act-D treatment. The relative half-life of the SPUD isoforms transcript was calculated from three independent samples. Errors represent the SD derived from three independent experiments.

Analysis of conserved regions within *CDKN1A* intron 1 searching for potential regulatory elements showed a region that was determined to be HuR binding sites using the RBP-predictor program RBPmap (49,50, Figure 3A). Consistent with the role of HuR regulating intronic/full-length ratios under stress conditions (51), analysis of the published CLIP-seq data indicated a peak both before and after stress for HuR at the predicted binding site in *CDKN1A* intron 1 (Supplementary Figure S2A). HuR can bind not only the 3′ untranslated region (3′UTR) of full-length mRNAs, regulating their stability (38,52), but also lncRNAs, affecting their stability and localization (53,54). In fact, intronic HuR sites are more prevalent than 3′UTR binding regions (55) RNA-immunoprecipitation (RIP) assays showed that HuR can form complexes with both *CDKN1A* full-length mRNA and ‘unspliced SPUD’ but not with SPUD (Figure 3B). HuR binding to *CDKN1A* full-length was previously described to be induced after UV treatment through a region in the 3′UTR (38). ‘Unspliced’ binding to HuR was also observed using nuclear extracts (NEs) with biotinylated *CDKN1A* isoforms in RNA-pull down (RPD) assays (Figure 3C-D). When NEs from cells recovered at different time points after UV treatment were used in RPD assays, HuR binding to ‘unspliced SPUD’ was not significantly modified (Figure 3E), except for a small but significant decrease at early time points after UV treatment. Interestingly, HuR depletion upon UV treatment abolished not only the previously described increase in *CDKN1A* full-length mRNA (38) but also induction of both ‘unspliced SPUD’ and SPUD (Figure 3F, Supplementary Figure S2B-C). As HuR is a highly abundant nuclear protein (56), overexpression of HuR did not affect the UV-induced increase in *CDKN1A* full-length mRNA, ‘unspliced SPUD’ and SPUD (Supplementary Figure S2D).

To further understand the mechanism for this HuR-mediated regulation of *CDKN1A* isoforms, HCT116 cells were pre-treated with UV with a 2 h recovery to induce APA followed by actinomycin D treatment for the time course indicated (Figure 3G). Intriguingly, HuR depletion resulted in ‘unspliced SPUD’ half-life decreased from 8.1 to 2.8 h. Notably, a downward trend in SPUD was observed upon HuR depletion, but this was not significant. Overall, these results indicate that HuR regulates the stability of ‘unspliced SPUD’, adding another lncRNA to HuR’s regulatory repertoire (13).

### SPUD is induced in cancer and normal cells under a variety of damaging conditions in a p53-dependent manner

**Figure 4.**
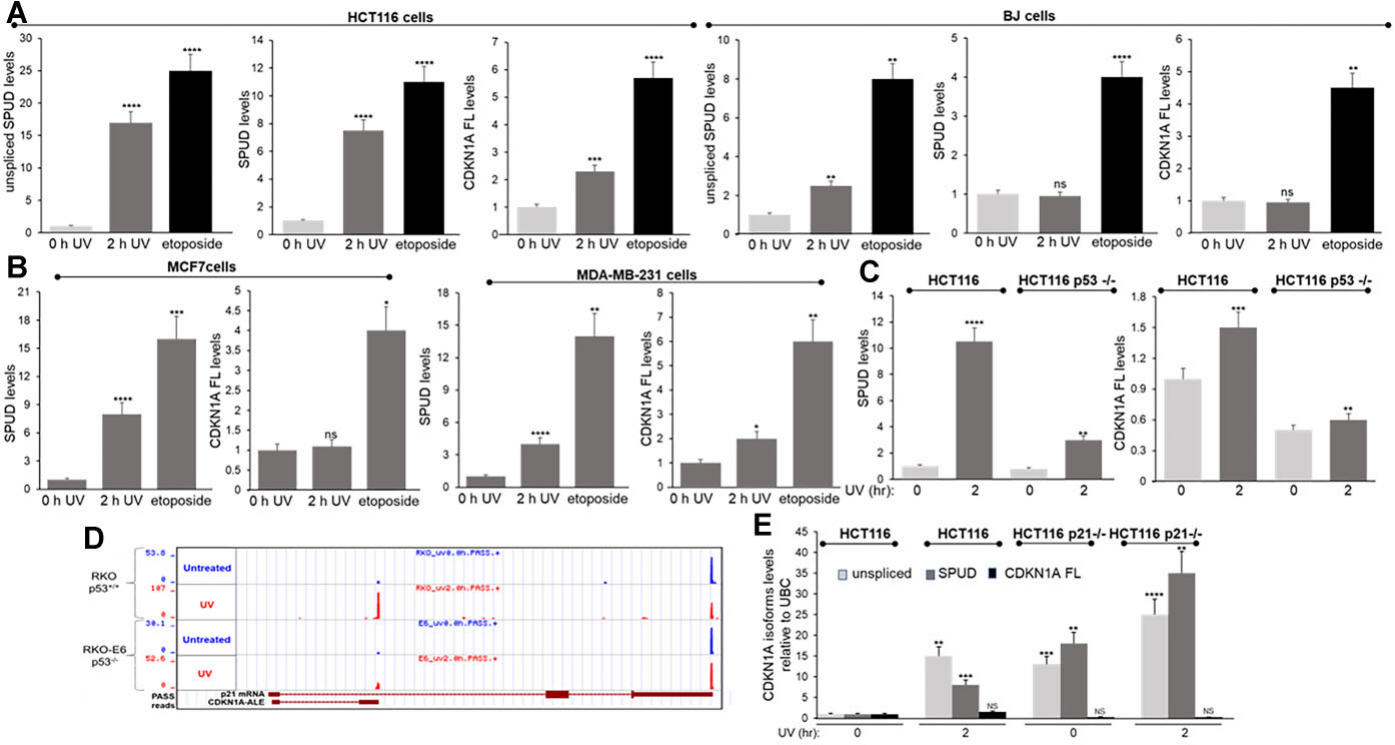
SPUD isoforms are expressed in a variety of cell lines and stressor conditions in a p53-dependent manner. **(A)** Induction of SPUD was higher in tumorigenic cell lines (HCT116) than into non-cancer cell lines (BJ-5ta immortalized foreskin fibroblasts). Total RNA was purified from the indicated cell lines and analyzed by qRT-PCR as in Figure 2A. Cells were treated with UV irradiation (20 J/m², 2 h recovery) or with etoposide (20 μM) 16 h prior to total RNA extraction. Data shown is from 3 independent biological samples analyzed by triplicate, SD (*n* = 3). ** *P* < 0.005, *** *P* < 0.0005, **** *P* < 0.00005. **(B)** MCF7 or MDA-MB-231 breast cancer cells were treated and analyzed as in (A). *P < 0.01, ** *P* < 0.005, *** *P* < 0.0005, **** *P* < 0.00005. **(C)** Loss of p53 significantly reduces expression of both SPUD isoforms and *CDKN1A* full-length. HCT116 and HCT116 p53^−/−^ cells were treated and analyzed as in (A). **(D)** 3′READS data indicates ablation of intron-APA in p53-null cells. Analysis of RKO and p53-null RKO-E6 colon carcinoma cell lines before (blue) and after (red) UV-mediated DNA damage. Detection was scored in Reads Per Million (RPM), calculated as the number of poly(A) site supporting (PASS) reads of hat site in a million unique PASS reads per sample. **(E)**. ‘Unspliced SPUD’ and SPUD expression is detectable and dysregulated in HCT116 p21^−/−^ cells. Both HCT116 and HCT116 p21^−/−^ cell lines were treated and analyzed as in (A).

We next extended our studies to other cell lines and analyzed the effect of different stressors. A delay in *CDKN1A* mRNA and p21 protein expression due to a block in transcription elongation has been described under certain stressors, such as UV and HU treatment (21–24), but not others, such as the strong p21 inducer, etoposide (57). Interestingly, expression of both SPUD and full-length mRNA was induced upon etoposide treatment (Figure 4A, B).

SPUD generated by UV-induced intron-APA in *CDKN1A* was first described in colon carcinoma RKO cells (34). Here we also show that SPUD can be detected in HCT116 colorectal carcinoma (Figures 1–3), a cell line historically used to study *CDKN1A* transcriptional regulation (19,24), and in keratinocytes (Supplementary Figure S3A). SPUD induction was also observed in MCF7 and MDA-MB-231 cancer cell lines, and immortalized skin fibroblast cell line BJ1-hTERT (Figure 4A-B, Supplementary Figure S3B). SPUD also responded in a dose dependent manner (Supplementary Figure S3C). These results suggest that UV-mediated induction of SPUD is a widespread, common feature of DDR in cells with different tissues of origin as shown in Figure 1B. This widespread expression has been shown for other lncRNAs (58).

Our previous 3′ READs data generated in RKO-E6 cells, which lack functional p53, indicated that many intron-APA events were directly or indirectly p53-dependent, including *CDKN1A* intron-APA (34). As SPUD is a sense-strand intragenic transcript of *CDKN1A* gene, we postulated that SPUD transcriptional induction might be subject to the same promoter regulation by p53 (59). Indeed, UV-induced expression of both SPUD and, as previously described (21–24,60), *CDKN1A* full-length decreased, but was not entirely abolished, in HCT116 p53−/− cells (Figure 4C). This is consistent with our previous high-throughput screen that showed the reduction in PAS supporting (PASS) reads is partly dependent on p53 (Figure 4D, 34). Notably, a previously derived cell line lacking p21 expression due to recombination-mediated excision of coding exons 2 and 3 (61,62) exhibited significantly higher SPUD levels at baseline as well as augmented UV-mediated induction of SPUD but not of *CDKN1A* full-length (Figure 4E). Together, these results indicate that SPUD is expressed across a range of cell-types and treatments is dependent on p53.

### SPUD is a lncRNA that regulates the translation of p21 protein affecting cell cycle

**Figure 5.**
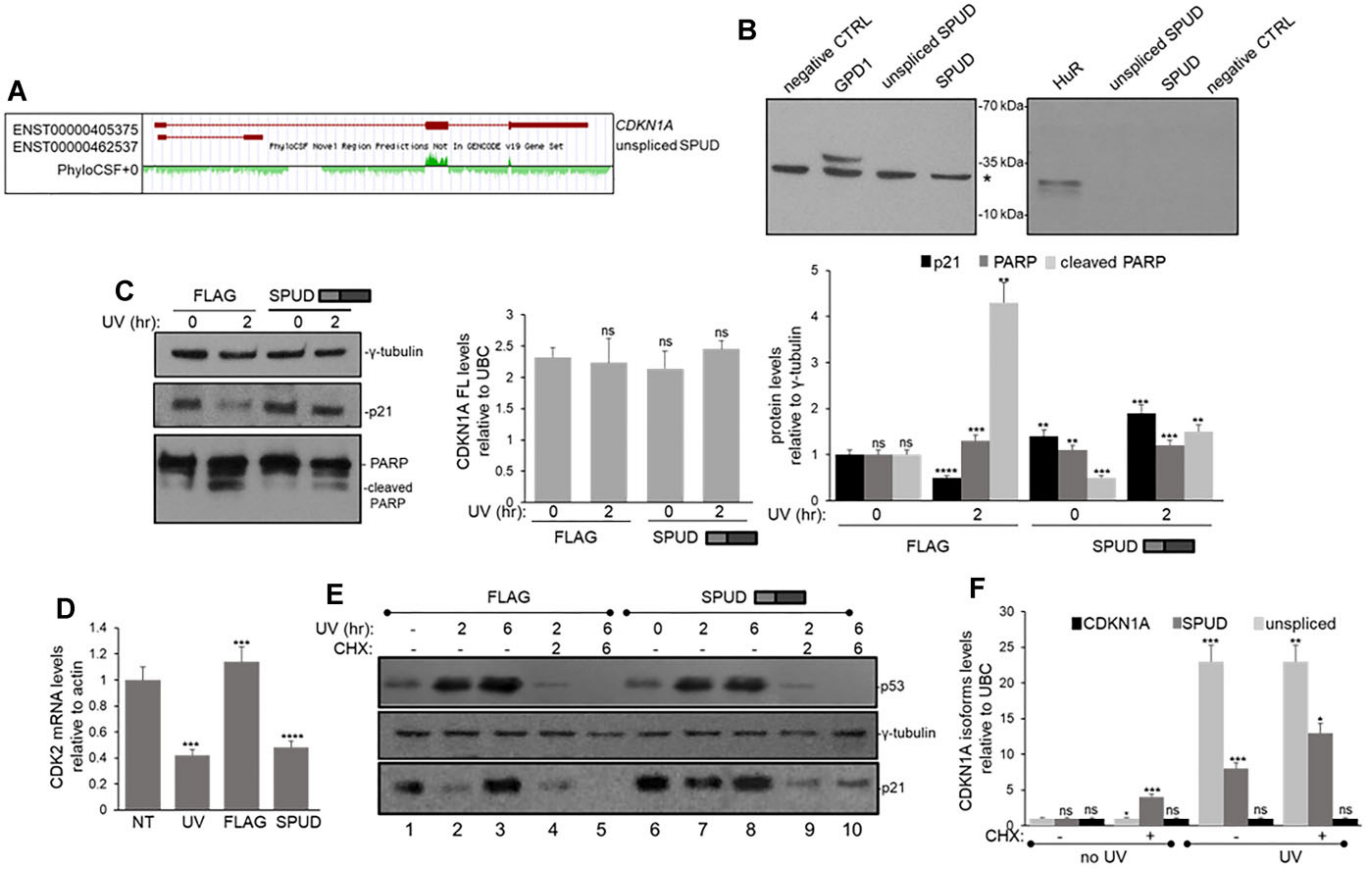
SPUD is a lncRNA that regulates p21 post-transcriptionally. **(A)** PhyloCSF shows negative average score for *CDKN1A* exon 1 and intron 1, whereas p21 exons 2 and 3 have positive score. UCSC genome browser screenshot of PhyloCSF analysis of *CDKN1A*. Positive green peaks indicate coding sequence conservation, negative peaks indicate no positive selection for coding sequence. **(B)***Ex vivo* and *in vitro* translation reactions yield no detectable protein product for both ‘unspliced SPUD’ and SPUD isoforms. Protein potentially derived from uATG of SPUD is expected to be ∼15 kDa. Left: overexpression of SPUD isoforms make no detectable protein in HCT116 cells. CMV-driven expression plasmids with N-terminal FLAG tags containing no transcript, GDP1 as positive control, or either ‘unspliced SPUD’/SPUD transcripts were overexpressed. Total protein was extracted from transfected cells and immunoassayed with anti-FLAG antibodies. (*) indicates non-specific band detected by the antibody. A representative gel from three independent samples is shown. Right: *In vitro* translation reactions yield no detectable protein for SPUD isoforms. T7-driven plasmids containing HuR as a positive control or ‘unspliced SPUD’/SPUD transcripts were *in vitro* translated in rabbit reticulocyte lysates. Translated proteins were labelled with biotinylated lysines, reactions were run on SDS-PAGE and immunoassayed against streptavidin-HRP conjugates. A representative gel from three independent samples is shown. **(C)** SPUD overexpression upregulates p21 and mitigates UV-mediated transient downregulation of p21 protein without affecting *CDKN1A* full-length mRNA levels. Cleaved PARP is detected as a biomarker for apoptosis. Left: HCT116 cells were transfected with CMV-driven expression plasmids with FLAG-tagged SPUD transcript and exposed to UV irradiation (20 J/m², 2 h recovery). NEs were immunoassayed for p21, PARP and γ-tubulin as control. Gel shown is representative of three independent biological samples. Middle: RNA from transfected HCT116 cells with expression constructs described above was analyzed by qRT-PCR for *CDKN1A* full-length mRNA and normalized to UBC. Right: Quantification of cleaved PARP. Data shown is from 3 independent biological samples analyzed by triplicate, SD (*n* = 3). ns: no significant, ** *P* < 0.005, *** *P* < 0.0005, **** *P* < 0.00005. **(D)** Overexpression of SPUD leads to CDK2 mRNA downregulation. HCT116 cells were treated as in **(C)** and analyzed for CDK2 transcript by qRT-PCR as in Figure 2A. **(E)** Inhibiting translation with CHX prevents the effect of overexpressed SPUD on the reduction in UV-induced decrease in p21, but has no effect on p53 levels. HCT116 cells were transfected as in (C) and treated with either UV (20 J/m^2^) or CHX (2 μg/ml)/UV (20 J/m^2^) for the indicated times. Samples were analyzed as in (C). **(F)** Inhibiting translation with CHX leads to higher SPUD induction after UV treatment. Cells were treated as in (D) for RNA purification analyzed by qRT-PCR for *CDKN1A* isoforms as in Figure 2A.

An intriguing aspect of *CDKN1A* full-length mRNA is that the coding sequence begins in exon 2 downstream of the APA signal (Figure 5A). The mRNA also has an inhibitory upstream open reading frame (uORF) ATG in exon 1 (42). PhyloCSF analysis of *CDKN1A* gene confirmed the positive selection (average score 4.89) for *CDKN1A* full-length mRNA ORF (Figure 5A,^63^,^64^), whereas average PhyloCSF score for SPUD-ALE in the same frame as uORF ATG was -4.43. The protein potentially derived from uATG of SPUD would be ∼15 kDa with the polyA signal as stop codon, whereas ‘unspliced SPUD’ had no discernible ORF. To test whether SPUD coded for protein, N-terminal FLAG-tagged SPUD was overexpressed in HCT116 cells in the uATG frame. While FLAG immunoblotting detected a band for the FLAG-GPD1 control, it did not identify a specific band for SPUD isoforms (Figure 5B, left). Additionally, *in vitro* translation of SPUD constructs was also negative (Figure 5B, right), while HuR positive control was detected. Thus, these results are consistent with the idea that SPUD is a putative intragenic lncRNA within*CDKN1A* gene.

Several notable examples of intragenic/intronic lncRNAs have been described working either *in cis* or *in trans* to affect the expression of the protein of their host protein-coding gene (5,65). Therefore, we examined the role of SPUD on p21 protein expression levels, knowing that a downregulation occurs at early time points after UV treatment (Figures 5C, D, 4). Interestingly, samples from cells overexpressing SPUD mitigated UV-induced downregulation of p21 expression (Figure 5C, left), and this was independent of *CDKN1A* full-length mRNA levels (Figure 5C, right; Supplementary Figure S3D). An upregulation of p21 was also observed when expressing the ‘unspliced SPUD’ plasmid (Supplementary Figure S3E), indicating that the exogenous transcript was sliced and transported to the cytoplasm. In addition, while UV-induction increased levels of cleaved PARP, which was shown to be inhibited by p21 expression (66), SPUD upregulation diminished PARP cleavage after UV treatment (Figure 5C). Likewise, expression of SPUD also decreased cyclin-dependent kinase 2 (Cdk2) mRNA (Figure 5D), a key p21 target in DDR whose inactivation affects the cell cycle (67). These results suggest that SPUD works *in trans* as a p21 translational regulator, which is also supported by its cytoplasmic localization as shown in Figure 2E, F. Consistent with this, treatment of cells with cycloheximide (CHX), a translational inhibitor, prevented the enhanced expression of p21 that occurred during exogenous induction of SPUD (Figure 5E, compare lanes 7–8 to 9–10). Neither the levels of SPUD or CDKN1A RNAs are significantly affected by CHX after UV treatment (Figure 5F), therefore, any effects on p21 protein can be explained at the levels of CDKN1A mRNA translation or p21 protein stability.

**Figure 6.**
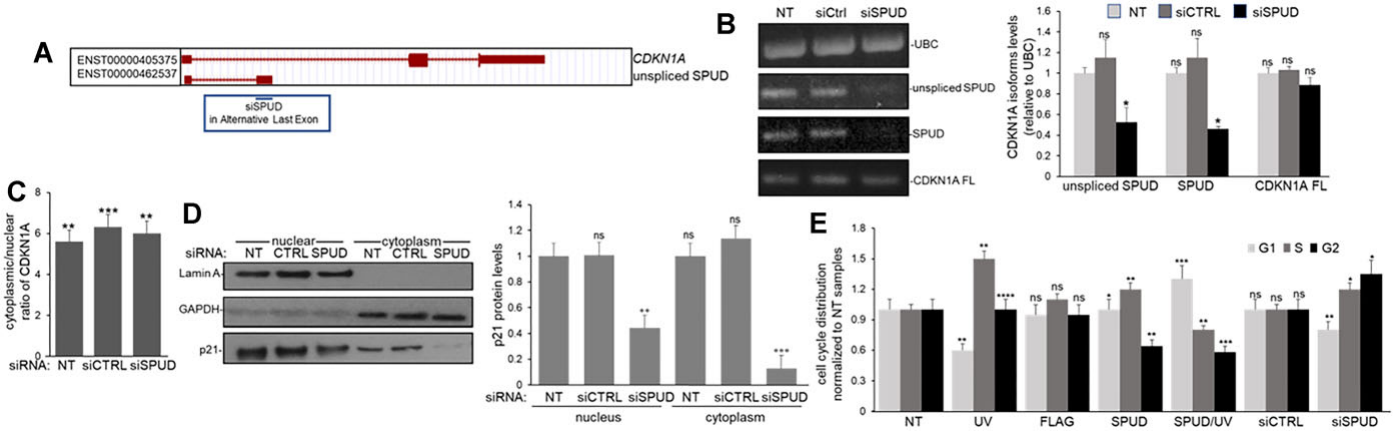
SPUD depletion decreases p21 protein expression without significantly affecting CDKN1A full-length mRNA levels. **(A)** Schematic of the design of an siRNA to target SPUD (siSPUD). Custom siSPUD was designed using siDESIGN Center (Horizon Discovery) targeting the entire APA exon as input. The black line represents the location of siSPUD in SPUD-ALE, the representation is not to scale. **(B)** siSPUD can deplete SPUD expression without significantly affecting CDKN1A full-length expression. RNA from HCT116 cells treated with siSPUD were analyzed by semi-quantitative PCR (left) or qRT-PCR (right). PCR products were separated on an agarose gel and detected by ethidium bromide staining. UBC amplification was used as loading control. Shown is a representative gel from three independent assays. qRT-PCR for CDKN1A isoforms was performed as in Figure 2A. Data shown is from three independent biological samples analyzed by triplicate (*n* = 3). ns; not significant; *P < 0.01. **(C)** Cytoplasmic/nuclear ratio of full-length CDKN1A mRNA does not change after siRNA mediated knockdown of SPUD. Nuclear and cytoplasmic RNA extraction was performed on SPUD depleted samples as in Figure 2E. Cytoplasmic/nuclear ratio of 2^−ΔΔCT^ of equal RNA input. U2 snRNA and GAPDH were used as fractionation controls. Data shown is from three independent biological samples analyzed by triplicate, (*n* = 3). **P < 0.001 and ***P < 0.0001. **(D)** SPUD depletion reduces the total abundance of cellular p21 protein. Left: Nuclear and cytoplasmic protein fractions were prepared from cells treated as in (B). Samples were analyzed by immunoblotting for p21 expression. Lamin A and GAPDH were used as fraction control. NT, non-transfected; CTRL, control siRNA. Right: SPUD knockdown has greater impact on cytoplasmic than nuclear p21. Data shown is from 3 independent biological samples analyzed by triplicate (*n* = 3). ns; not significant; **P < 0.001 and ***P < 0.0001. **(E)** Altering SPUD expression affects cell cycle distribution. Cell cycle distribution of different cells were normalized to non-treated (NT) cells as a fold change in the percentage of cells in each cell cycle stage. HCT116 cells were treated for SPUD overexpression as in Figure 5 or for SPUD depletion by siRNA-mediated knockdown as in (B). Cells were fixed with 4% formaldehyde, permeabilized with 95% ethanol and then treated with propidium iodide (PI) for FACS analysis. Data shown is from 3 independent biological samples analyzed by triplicate (*n* = 3). ns; not significant; *P < 0.01; **P < 0.001; ***P < 0.0001 and ****P < 0.00001.

To confirm the results seen for overexpression, depletion of SPUD and ‘unspliced SPUD’ using an siRNA targeting the APA exon (siSPUD, Figure 6A-C, ∼60% decrease) did not significantly change the levels and cellular distribution of *CDKN1A* full-length mRNA (Figure 6B, C) but did decrease p21 protein levels in both cytoplasmic and nuclear fractions (Figure 6D). 5′RACE results (Supplementary Figure S3F) were similar to the ones obtained with a 5′ forward primer in exon 1 (Figure 6B) for both CDKN1A full-length and SPUD upon siRNA treatment, indicating that siSPUD can deplete SPUD expression without significantly affecting the 5′UTR of cytoplasmic CDKN1A full-length. Together, our data indicate that the decrease in p21 protein levels was due to the knockdown of SPUD.

As previously described (68), flow cytometry analysis of UV-treated cells showed an increase in S-phase and a decrease in G1 due to p21 decrease (Figure 6E). Overexpression of SPUD under UV treatment led to an increase in G1 and decrease in S/G2 phase, as expected for induction of p21 protein expression, whereas SPUD depletion resulted in decrease in G1 and increase in S/G2, consistent with p21’s role as a cell proliferation inhibitor (69). In fact, SPUD depletion increased cell count compared to siCtrl treated cells (Supplementary Figure S3G), indicating a loss in cell-cycle arrest capabilities. Together, these results indicate that SPUD has a positive regulatory role on p21 translation and cell cycle progression.

### Different SPUD isoforms associate with ribosome

**Figure 7.**
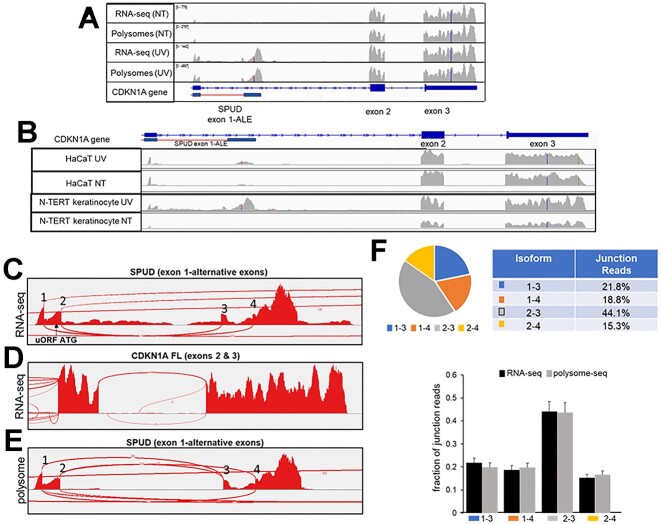
SPUD associates with polysomes as multiple isoforms after UV treatment in cell lines. **(A)** Read coverage tracks from RNA-seq and polysome-seq data obtained from N-TERT keratinocytes (GSE99745) before (NT) and after UV treatment (40 J/m^2^) were analyzed at *CDKN1A* gene using IGV viewer (hg38). The location of SPUD is indicated with a red line. **(B)** Read coverage tracks of RNA-seq data from HaCaT (GSE198792) and N-TERT cells before and after UV treatment. **(C)** Sashimi plot for the splice junctions of SPUD after UV treatment in N-TERT keratinocytes (GSE99745). The location of *CDKN1A* mRNA uORF ATG is indicated. **(D)** Sashimi plot showing one predominant splice junction for *CDKN1A* mRNA coding exons. **(E)** Sashimi plot of polysome-seq showing the presence of four isoforms of SPUD in the polysome pellet. **(F)** Top:SPUD in RNA-seq data (*n* = 3). Bottom: quantification of the fraction of each SPUD isoform in RNA-seq and polysome-seq samples according to the junction reads from the Sashimi plot in (C) and (E). Shown are the results from the three biological replicates available.

We next determined whether SPUD is detected in high-throughput data of UV treated and non-treated cells. Analysis of a pre-existing RNAseq dataset of UV-treated N-TERT (100 J/m^2^, Figure 7A, B; 41) and HaCaT (Figure 7B) keratinocytes showed a strong induction of SPUD after 8 h of UV-B treatment relative to full-length. While the cell lines and stress conditions are different, these results are consistent with the strong induction beyond baseline detected for SPUD 10 h post-UV in HCT116 cells as shown in Figure 2A. Surprisingly, splice junction coverage of SPUD after UV revealed four predominant spliced isoforms (1–3, 1–4, 2–3, 2–4; Figure 7C), whereas only one junction between exons 2 and 3 was detected for *CDKN1A* full-length (Figure 7D). Notably, isoform 2–4 corresponded to the SPUD isoform characterized in the previous figures.

Both ribosome and polysome profiling (70,71) studies have highlighted that approximately half of the annotated lncRNAs localize in the cytoplasm, many of which are associated with ribosomes. As shown in Figure 7A, Investigation of polysome-associated RNAs from the same N-TERT study (42) showed that both SPUD and *CDKN1A* full-length could be detected associated with polysomes. Accordingly, the same four SPUD spliced isoforms as shown in Figure 7C were present in the polysome pellet (Figure 7E); however, intronic reads present in ‘unspliced SPUD’ were absent, consistent with the nuclear localization of this precursor as shown in Figures 2E, F. Notably, the percentage of each isoform was equivalent between RNAseq and polysome-seq datasets (Figure 7F), suggesting lack of enrichment for any particular isoform in polysomes. Importantly, only isoforms 2–3 and 2–4 contained the uORF ATG from *CDKN1A* mRNA (42,72). Neither isoform 1–3 or 1–4 contained ORFs > 6aa, suggesting that SPUD may be ribosome-associated without participating in active translation for a specific ORF.

### RBPs CUGBP and CRT competitively bind both SPUD and *CDKN1A* full-length

**Figure 8.**
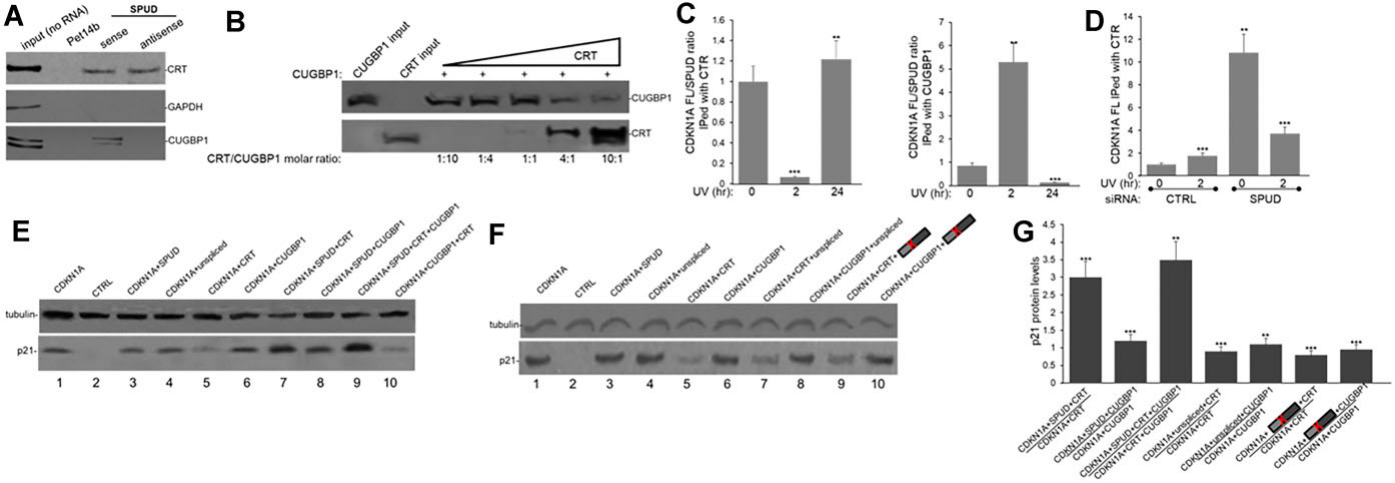
RBPs CUGBP and CRT bind directly to SPUD to regulate p21 translation. SPUD depletion results in an increase in CRT-*CDKN1A* full-length binding. **(A)** CUGBP and CRT bind directly to SPUD. Biotinylated sense and antisense SPUD were used in RPD assays with HCT116 cell lysate. Pet14b plasmid-generated RNA was used as a negative control. 5% input from HCT116 cell lysate is shown. As CRT binds a stem loop in exon 1, binding to sense and antisense is expected. CUGBP1 binds sequence specific, so only binding the sense is expected. GAPDH was used as loading control in Western blot analysis. A representative gel from three independent biological samples analyzed by triplicate is shown. **(B)** CUGBP and CRT compete for binding to SPUD. GST-CUGBP1 bound to biotinylated SPUD was mixed with increasing amounts of GST-CRT in RPD assays. Western blot analysis for the pulldown proteins is shown. A representative gel from 3 independent biological samples analyzed by triplicate is shown. **(C)** CRT binding to SPUD is favored over CDKN1A full-length binding at early times in the DDR, whereas CUGBP1 binding to CDKN1A full-length is favored over SPUD binding in non-treated cells and later in DDR. RNA-IP assay using either CRT, CUGBP1 or IgG antibody and samples from crosslinked HCT116 cells treated with UV irradiation (20 J/m^2^, recovery time indicated). Crosslinking was with 0.1% formaldehyde prior to sonication and immunoprecipitation. qRT-qPCR was performed on IP’ed transcripts. The CDKN1A/SPUD ratios observed in each condition were normalized to the values of non-treated samples. Data shown is from three independent biological samples analyzed by triplicate, SD (*n* = 3).**P < 0.001 and ***P < 0.0001. **(D)** RNA-IP performed as in (C) with samples of HCT116 cells depleted in SPUD. Samples were analyzed as in (C). **(E–G)** CRT inhibits *CDKN1A* full-length translation, and this is rescued by the addition of SPUD but not of ‘unspliced SPUD’ or SPUD stem-loop mutant. *CDKN1A* cDNA was transcribed by T7 polymerase from human *CDKN1A* gene expression plasmid. *In vitro* transcribed SPUD, ‘unspliced SPUD’ or SPUD stem-loop mutant and/or recombinant GST-CUGBP1 and GST-CTR were added to *in vitro* translation assays in rabbit reticulocyte lysate. Samples were analyzed by Western blot as in Figure 6D. A representative gel from three independent biological samples analyzed by triplicate is shown. (G) Quantification of p21 levels in the *in vitro* translation assays shown in (E-F). Errors represent SD (*n* = 3). **P < 0.001 and ***P < 0.0001.

Due to the apparent *in trans* translational regulatory function of SPUD (Figures 5 and6), as well as the finding that SPUD localizes to polysomes (Figure 7), we decided to investigate the potential functional overlapping of SPUD with other known translational regulators of p21. Most RBPs bind *CDKN1A* mRNA in the 3′UTR, except for CRT and CUGBP1 (73,74). CRT and CUGBP1 compete for binding in exon 2 of *CDKN1A* mRNA upstream of canonical ATG and have opposing effects on p21 translation and on cell proliferation through p21 expression (73). While CUGBP1 activates p21 translation (73,75), CRT blocks translation by stabilizing a stem–loop in exon 2 of *CDKN1A* full-length (73). ViennaFold (76) predicted the presence of intramolecular stem-loop in SPUD-ALE (Supplementary Figure S4A) similar to the one described in exon 2 (73). Using *in vitro* biotinylated SPUD, RPD assays with HCT116 cell lysates showed that both CRT and CUGBP1 can bind SPUD (Figure 8A), with CRT binding to both sense and antisense strands probably to the intramolecular stem–loop predicted in SPUD-ALE (Supplementary Figure S4A). CUGBP1 binds sequence specific, therefore it binds only the sense strand. A deletion of the loop located in SPUD-ALE (ΔGGCCG derivative), which is not present in the *CDKN1A* full-length transcript, abolished the SPUD-mediated induction of p21 protein level (Supplementary Figure S4B), independent of SPUD and *CDKN1A* full-length mRNA levels. As with the CDKN1A full-length transcript (73), our RPD assays using limiting amounts of GST-CUGBP1 and increasing molar concentrations of GST-CRT showed that CRT and CUGBP1 competitively bind to SPUD (Figure 8B). Importantly, at 1:1 molar ratio, SPUD preferentially bound CUGBP1, whereas p21 preferentially bound CRT (73).

To further analyze SPUD interaction with CRT and CUGBP1 RIP assays were performed using antibodies against each protein and samples from HCT116 cells exposed to UV treatment. Early in UV response (2 h), CRT preferentially bound SPUD over *CDKN1A* full-length and CUGBP1 preferentially bound full-length over SPUD, returning to basal levels after 24 h (Figure 8C). The expression levels of both CRT and CUGBP1 did not change during the DDR progression (Supplementary Figure S4C) Surprisingly, when the RIP assays were repeated after SPUD depletion, the binding of CRT to *CDKN1A* full-length increased both in stress and non-stress conditions (Figure 8D), suggesting competitive interaction of SPUD and full-length with CRT and providing a mechanism through which p21 translation might decrease. Notably, pre-treatment of cells with CHX to inhibit translation led to a significant increase in SPUD levels in all the conditions tested (Supplementary Figure S4E) without an increase in p21 protein levels (Supplementary Figure S4D), supporting the idea that SPUD-mediated regulation of p21 protein levels occurs through active translation.

To test the role of SPUD on *CDKN1A* full-length translation we analyzed the effect of SPUD ‘unspliced SPUD’, CRT and CUGBP1 on in *in vitro* translation using reticulocyte lysates (Figure 8E–G). Analysis of the proteins used in these assays is shown in Supplementary Figure S4F. As previously described (73), the addition of CRT inhibited p21 translation (Figure 8E, compare lanes 1 and 5), and this was rescued by the addition of SPUD (lane 7) but not of ‘unspliced SPUD’ (Figure 8F, compare lanes 1, 5 and 7). The addition of CUGBP1 did not significantly affect p21 translation (Figure 8E, compare lanes 1 and 6), and this was not changed by the addition of either SPUD (lane 8) or ‘unspliced SPUD’ (Figure 8F, compare lanes 1, 6 and 8). The addition of either SPUD (Figure 8E, lane 3) or ‘unspliced SPUD’ (Figure 8F, lane 4) alone did not have a significant effect of p21 expression. When equimolar quantities of CRT and CUGBP1 were added to the reaction a significant decrease in p21 translation was detected (Figure 8E, lane 10); and this decrease was reversed by the addition of SPUD (lane 9). Neither the unspliced SPUD nor SPUD stem-loop mutant were able to reverse the CRT inhibition of CDKN1A translation (Figure 8F), consistent with CRT binding to the intramolecular stem-loop predicted in SPUD-ALE (Supplementary Figure S4A). Fold change in p21 protein levels by addition of SPUD are shown in Figure 8G. Together, this data with results shown in Figure 7 support our model that SPUD-associated with polysomes after UV treatment regulates p21 expression. These results are consistent with previous work (73) that showed that while a minor portion of total CRT is present in polysomal fractions, only polysome-associated CRT but not free CRT binds *CDKN1A*.

## Discussion

Cellular response to stress is achieved by the dynamic flux in gene expression. Post-transcriptional regulation of coding and non-coding RNA offers a fast method of adapting to a changing cellular environment. As intron-APA can alter the sequence composition of gene transcripts via intron retention and ALE inclusion (26), this alternative processing activation has the potential to diversify the functional output of genes or fine-tune the regulation of the canonical protein by the expression of additional proteins or non-coding RNAs (5). Therefore, understanding the complex interplay between APA events and genes involved in DDR/tumor suppression, such as *CDKN1A*/p21, can inform us about novel mechanisms to regulate cell-cycle checkpoints and the cell-fate decision between senescence and apoptosis during the progression of DDR. p21 plays a key role regulating cell fate (77–81), therefore changes in p21 expression levels and/or duration of this induction may be consequential for cell function. p21 has been described as a dose-sensitive ‘goldilocks protein’ which can be oncogenic at low or high levels, playing a key factor in chemotherapy effectiveness by inducing the cells to either enter senescence or maintain a proliferative state (19,20). Therefore, *CDKN1A* gene expression is tightly regulated (79); early in DDR, despite the presence of p53 at the promoter, there is a delayed in p21 expression upon stresses like UVC or HU (19,20), partially due to a block in transcription elongation somewhere in intron 1 (21–24). The fate of the truncated CDKN1A transcript generated in these conditions has not been investigated.

**Figure 9.**
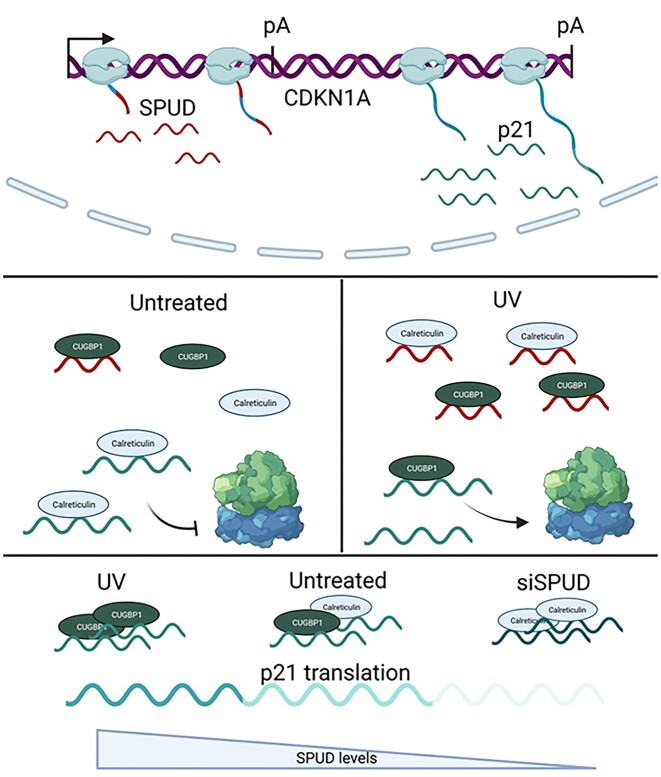
Working model involving CUGBP1 and CRT proteins functionally interacting with either *CDKN1A* full-length or SPUD. In non-stress conditions (left), SPUD levels are low and *CDKN1A* full-length mRNA is bound by CRT, which is a repressor of p21 translation. After DNA damage (right), SPUD is induced, stabilized by HuR, spliced and then transported to the cytoplasm where it can sequester the negative regulator CRT. Then *CDKN1A* full-length transcripts can bind the activator of translation CUGBP1 resulting in induction (upwards arrow) of p21 expression and proper progression of DDR.

In this study, we provided evidence that promoter-proximal intron-APA and alternative splicing within intron 1 of *CDKN1A* generate an ALE-containing lncRNA SPUD (Figures 1–2) under a variety of damaging conditions (Figure 4). *CDKN1A* intron 1 APA was first detected not only in our study of widespread APA occurrence in introns, biased towards the 5′ end of genes, after UV-treatment (34) but was also detected via EST and via GTEx (Figure 1A, B). SPUD has low abundance compared to *CDKN1A* full-length isoform under basal conditions; SPUD is localized in the cytoplasm, is stable, and induced in cancer and normal cells during DDR (Figures 2 and4). The RBP HuR binds to and promotes the stability of the unspliced precursor of SPUD (Figure 3). SPUD is a putative lncRNA regulated by p53 (Figures 4 and5). SPUD induction increases p21 but not *CDKN1A* full-length mRNA levels, affecting p21 functions in cell-cycle, CDK2 expression and cell growth (Figures 5 and6). Consistent with SPUD’s role on the induction of p21 expression at the translational level under stress conditions, SPUD is associated with polysomes (Figure 7) and rescues *CDKN1A* translation in the presence of the p21 translational inhibitor CRT (Figure 8). SPUD binding to CRT increases at early times after UV treatment (Figure 8) and provides a mechanism for enhanced p21 translation following transient decrease in *CDKN1A* full-length levels during S-phase and upon UV damage (82). Accordingly, SPUD depletion increases the level of CRT associated to *CDKN1A* full-length mRNA (Figure 8). Taken together, our results indicate that intron-APA within *CDKN1A* produces a lncRNA that can fine tune the expression of p21 during DDR and cell-cycle described in the working model in Figure 9. We propose that CUGBP1 and CRT proteins functionally interact with either *CDKN1A* full-length or SPUD. In non-stress conditions, SPUD levels are low and *CDKN1A* full-length mRNA is bound by CRT (Figure 8) in the polysome (Figure 7, 73), which is a repressor of p21 translation. After DNA damage, SPUD is induced and can sequester the negative regulator CRT. Then *CDKN1A* full-length transcripts can bind the activator of translation CUGBP1 resulting in induction of p21 expression and proper progression of DDR. LncRNAs are known to sequester proteins (83,84) and to act as endogenous competitors to mRNAs as they share RBP binding sequences (85). For example, a sense-lncRNA can regulate translation of its cognate mRNA, PABPN1, by sequestering a translational repressor (86).

Based on our results, we propose a model whereby intron-APA site activation and SPUD induction correlates with the previously described decrease in U1 snRNA levels during DDR (87), and this is reversible by U1 snRNA overexpression (34). Consistent with this, UV-induced activation of PAS in intron 1 of *CDKN1A* occurs at times of UV-mediated depletion of U1 snRNA (34) and decrease in APA and ‘unspliced SPUD’ levels after UV occurs at times U1 snRNA levels recover (>10 h after UV). The delayed induction of *CDKN1A* full-length, but not of the lncRNA SPUD after UV treatment, might indicate that the proximal and canonical PAS can be recognized independently. It is unlikely that *CDKN1A* undergoes sequential cleavage on the same molecular transcript (88), as both full-length mRNA and SPUD share exon 1. Consistent with studies that showed that canonical PAS can enhance proximal-splice site usage (89), our results showed that the UV-induced increase in ‘unspliced SPUD’ occurs prior to SPUD induction indicating that APA occurs prior to 3′ splice site recognition. Interestingly, a measurable subset of SPUD precursor appears to remain unspliced (Figure 7C).

Surprisingly, tandem PAS are in close proximity (∼30 nt) within *CDKN1A* intron 1 (Figure 2A). Tandem PAS are usually located within the 3′UTR with a median distance between PAS of ∼300 nt (90). Our 3′RACE results suggest that the first PAS encountered (ATTAAA) is preferentially used both before and after UV treatment (Figure 2D), whereas the downstream PAS (AATAAA) was used with less efficiency (and only after UV treatment). The region around the SPUD-ALE itself has also been shown to be a hub for binding activity of different factors; such as the transient binding of TGF-b-induced SMAD2/3 (91), MEF2C/D (92) and p21-specific splicing regulator SKIP (57). The retention of multiple PAS and the functional association of different factors support the presence of these signals playing an important role.

Once ‘unspliced SPUD’ precursor is spliced and exported to the cytoplasm, high levels of SPUD are detected at later time points after UV treatment as results of its high stability during DDR (Figures 2 and 3). The regulatory role of HuR on *CDKN1A* intron-APA and on the stability of the SPUD precursor is supported by previous studies that show the presence of HuR binding sites in the proximity of ALEs and APA signals by high-throughput and individual examples (51,93,94). The predicted HuR binding site in ‘unspliced SPUD’ overlaps with a highly conserved region within *CDKN1A* intron 1; HuR binds specifically to the ‘unspliced SPUD’, but not SPUD the cytoplasmic transcript, both before and during the progression of UV-mediated DDR (Figure 3B-E). HuR’s regulatory role appears to be independent from the APA event itself, as HuR depletion under transcription inhibition conditions resulted in a decrease in the half-life of ‘unspliced SPUD’, but not SPUD, following UV induction (Figure 3F, G). SPUD levels did not change significantly by HuR depletion/actinomycin D treatment, which we attribute to the high stability of the transcript and suggesting that HuR does not play a role in splicing or SPUD stability.

PhyloCSF analysis of *CDKN1A* gene confirms the positive selection for *CDKN1A* full-length mRNA ORF but not for SPUD (Figure 5A), supporting the role of SPUD as a lncRNA. This is further strengthened by the lack of *in vitro* translation of SPUD transcript or detection following expression of FLAG-tagged transcript beginning at exon 1 uORF ATG (Figure 5B). Furthermore, the multiple SPUD isoforms detected via existing RNA-seq possess similar polysome association, despite the presence of *CDKN1A* mRNA uORF in only a subset of the isoforms (Figure 7F).

SPUD is a positive regulator of p21’s, but not of p53’s, protein translation (Figure 5E). We found that SPUD competes with *CDKN1A* full-length for binding to two RBPs that competitively regulate p21 translation: the repressor CRT and the activator CUGBP1 (Figure 8A, B; 73). Our results show that loss of SPUD increases the association of CDKN1A full-length with CRT, and that CRT inhibition of p21 translation (73) is reverted by the addition of SPUD but not of ‘unspliced SPUD’ (Figure 8). SPUD is detected associated with polysomes (Figures 7E-F), which is consistent with previous studies showing translational regulation of p21 in polysomal fractions enriched in CRT-associated to *CDKN1A* (73). While a minor portion of total p21 translational inhibitor CRT is present in polysomal fractions, only polysome-associated CRT binds *CDKN1A* (73). Polysome association of lncRNAs to regulate translation of other mRNAs has been observed previously, including through complementary base-pairing to short sequences (13,71,95). We did not find any complementary sequences between SPUD and full-length mRNA. Interestingly, SPUD preferentially binds to CUGBP1 (Figure 8B; 1:1 ratio), whereas *CDKN1A* full-length preferentially binds to CRT (73) under non-stress conditions, suggesting that high levels of SPUD are necessary to remove CRT from *CDKN1A* to allow p21 translation upon stress conditions. While future experiments are necessary to further understand the mechanism behind SPUD regulation of p21 translation, it will also be interesting to establish whether SPUD plays additional cellular roles particularly in a p21 null background where SPUD is still expressed (Figure 4E) and dysregulation in protein expression and cellular functions have been described (61,62).

Altogether, our data have provided evidence of a sense-strand intragenic lncRNA produced within *CDKN1A* that functions *in trans* to fine-tune p21 expression during DDR, reinforcing the functional interaction of lncRNAs and cell-cycle genes. Understanding the complex interplay between intron-APA events and canonical gene products involved in DDR/tumor suppression, such as SPUD/p21, might help us in identifying mechanisms that drive tumor progression, relapse and treatment resistance.

## Supplementary Material

gkad899_Supplemental_File

## Data Availability

The data underlying this article are available in the article and in its online supplementary material.
