# Supplementary material for: Long non-coding RNA generated from CDKN1A gene by alternative polyadenylation regulates p21 expression during DNA damage response
Source: Nucleic Acids Res. 2023 Oct 23;51(21):11911–26. doi: 10.1093/nar/gkad899 (PMC10681730; doi:10.1093/nar/gkad899)
Supplement: gkad899_Supplemental_File [file gkad899_supplemental_file.pdf]

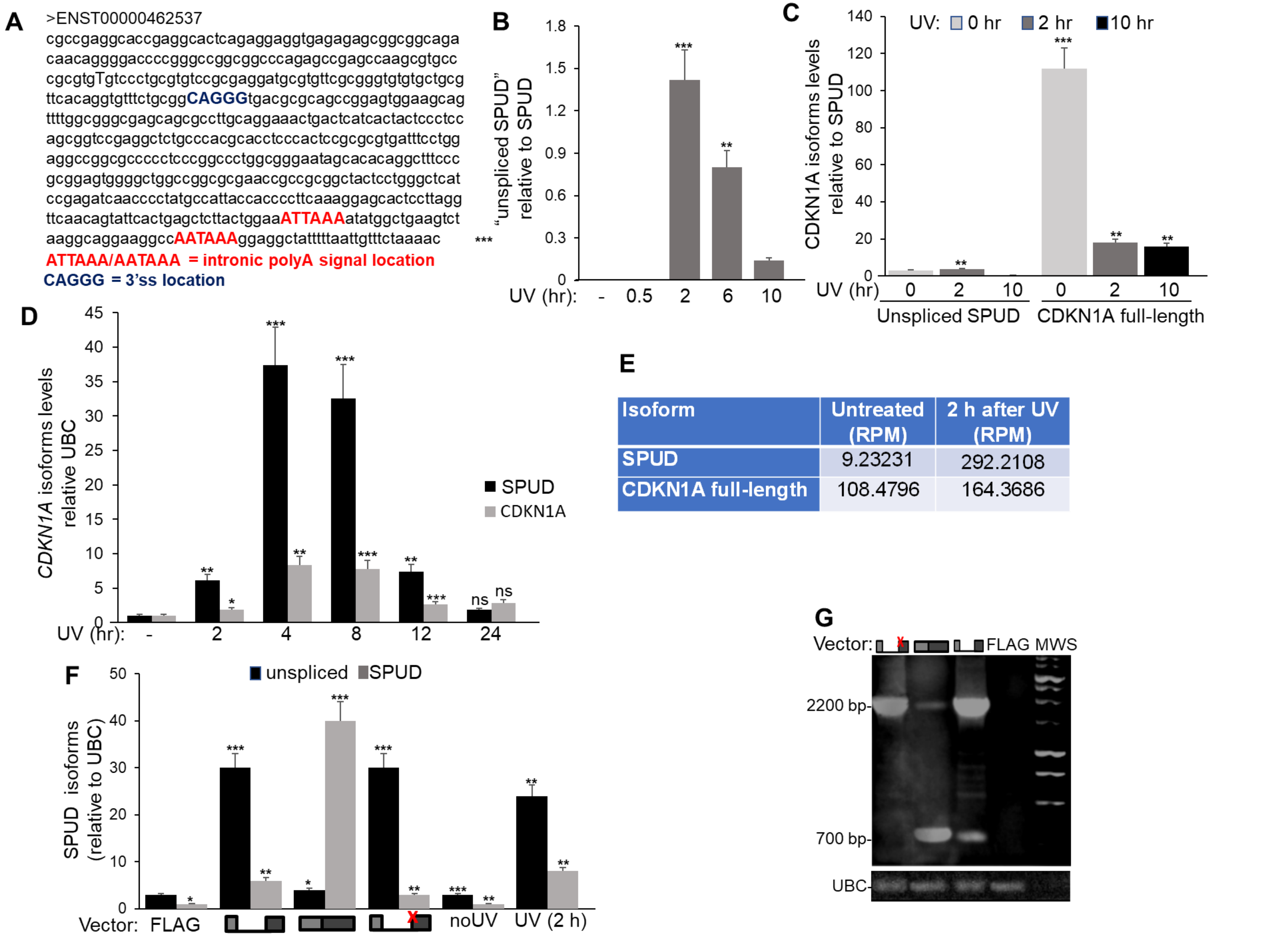

Isoform	Untreated (RPM)	2 h after UV (RPM)
SPUD	9.23231	292.2108
CDKN1A full-length	108.4796	164.3686

Supplementary Figure 1: Conserved elements within *CDKN1A* intron 1 corresponding to splicing and PAS. **(A)** Complete RNA sequence for ENST00000462537 (SPUD). Highlighted in dark blue is the putative 3’ss. Highlighted in red are the tandem canonical PAS. **(B)** Ratio of “unspliced SPUD” to SPUD calculated from semi-quantitative analysis from Figure 1D. HCT116 cells were treated with UV (20 J/m^2) and allowed to recover for indicated time points followed by RT-PCR using purified RNA and primer sets described in Figure 1C. cDNA was prepared using oligo(dT) primers. Values were normalized to non-treated cells. Errors represent SD (n=3). **P<0.001 and ***P<0.0001. **(C)** Difference between SPUD and *CDKN1A* full-length is reduced after UV treatment relative to each qRT-PCR amplicon. The ratios calculated for each condition were normalized to the ratios calculated in non-treated cells. **P<0.001 and ***P<0.0001. **(D)** Time course induction of SPUD and *CDKN1A* full-length after UV exposure (20 J/m^2) and the indicated recovery times. SPUD levels are induced at early points after UV treatment relative to samples from non-treated cells, reaching a max between 4-8 h going to unstressed levels after 24 h. While *CDKN1A* full-length induction follows a similar pattern with a delay at 2 h, the magnitude of the induction relative to non-stress conditions is lower. **(E)** Quantification of SPUD *versus* *CDKN1A* full-length transcript during DDR. Reads per million (RPM) values were determined as described in Figure 1A (34) for usage of intronic and 3’UTR PAS in *CDKN1A*. Data used in the pie charts shown in Figure 2B. **(F)** Comparison of the difference between *CDKN1A* isoforms overexpression with UV-treated cells relative to housekeeping gene. Values shown here were used in bar graph in Figure 2C. HCT116 cells were transfected with the indicated plasmids for 48 h prior to RNA extraction and qRT-PCR for *CDK1NA* isoforms. Three independent biological samples analyzed by triplicate is shown, SD (n=3). *P<0.01, **P<0.001 and ***P<0.0001. **(G)** Semi-quantitative RT-PCR analysis done as in (E) but replacing the forward primer for one located in the FLAG region, avoiding the detection of endogenous SPUD isoforms. Isoform distinction is based on molecular weight by gel electrophoresis. The results are similar to those obtained with exon 1 (panel E) forward primer due to the overexpression of the vectors. A representative gel from three independent assays from three biological samples is shown (n=3).

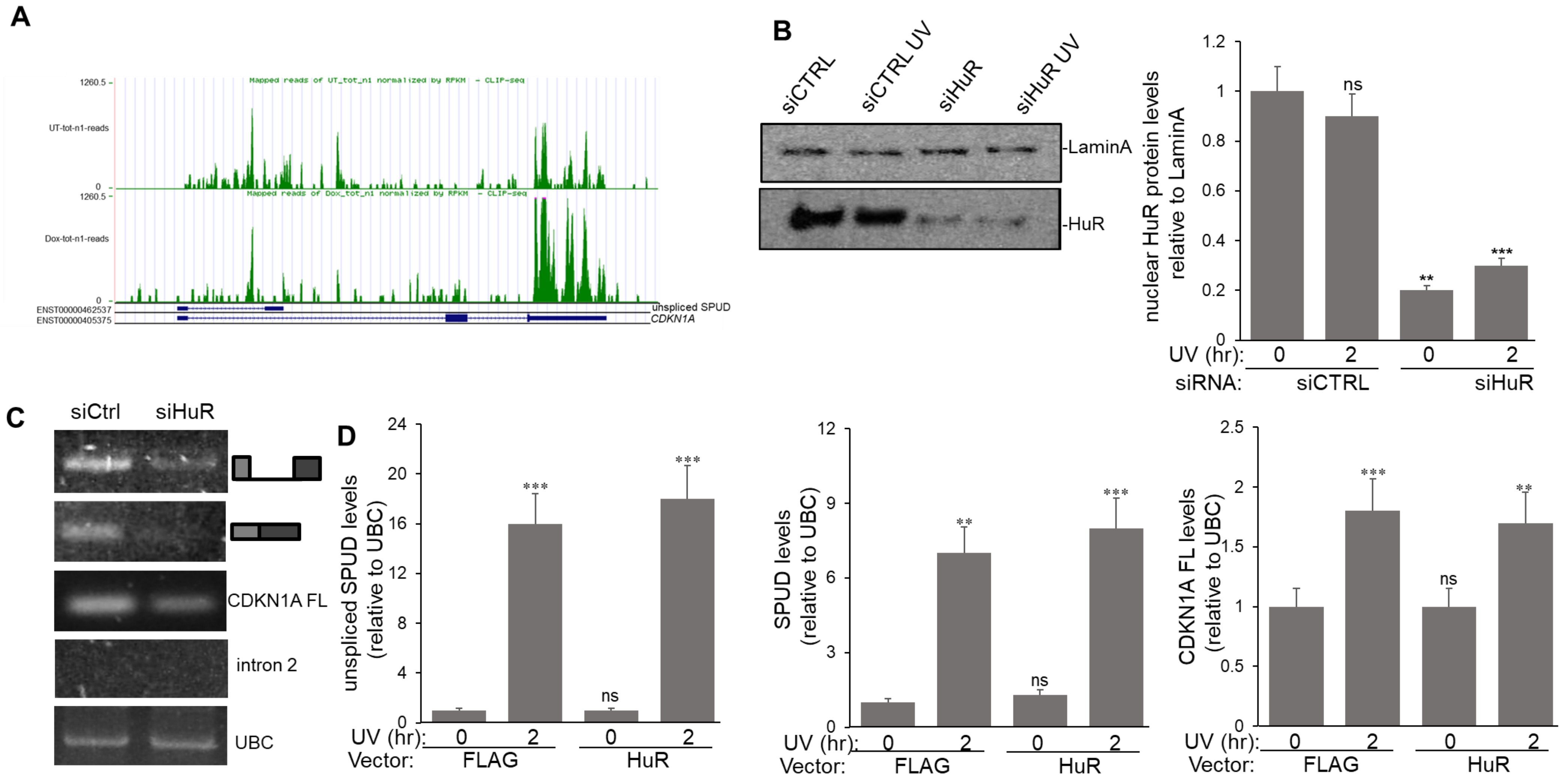

Supplementary Figure 2: (A) HuR CLIP-seq reads (51), showing strong HuR peak within SPUD intron in both untreated (UT) and stressed (Doxorubicin; Dox) cells. Blue boxes and lines at the bottom indicate exons and introns, respectively, of SPUD and *CDKN1A* full-length transcripts. **(B)** Knockdown of HuR using siRNA. siRNA against HuR was transfected into HCT116 cells for 48 h followed by RNA extraction or 2 h UV treatment then extraction. Left, NEs were immunoblotted for HuR and LaminA as loading control. A representative gel from three independent assays from three biological samples is shown (n=3). Right, quantification of immunoblot (n=3). **(C)** siRNA against HuR leads to loss of CDKN1A isoforms. RNA extracted from HuR knockdown samples underwent semi-quantitative RT-PCR and agarose gel analysis for CDKN1A isoforms. UBC amplification was used as loading control; CDKN1A intron 2 was used as control for genomic DNA contamination. A representative gel from three independent assays from three biological samples is shown (n=3). **(D)** HuR overexpression does not impact CDKN1A isoforms. Mammalian expression vector containing HuR cDNA was transfected into HCT116 for 48 h followed by qRT-PCR analysis for indicated transcripts. Shown are the results from three independent assays.

Murphy et al., Figure S3

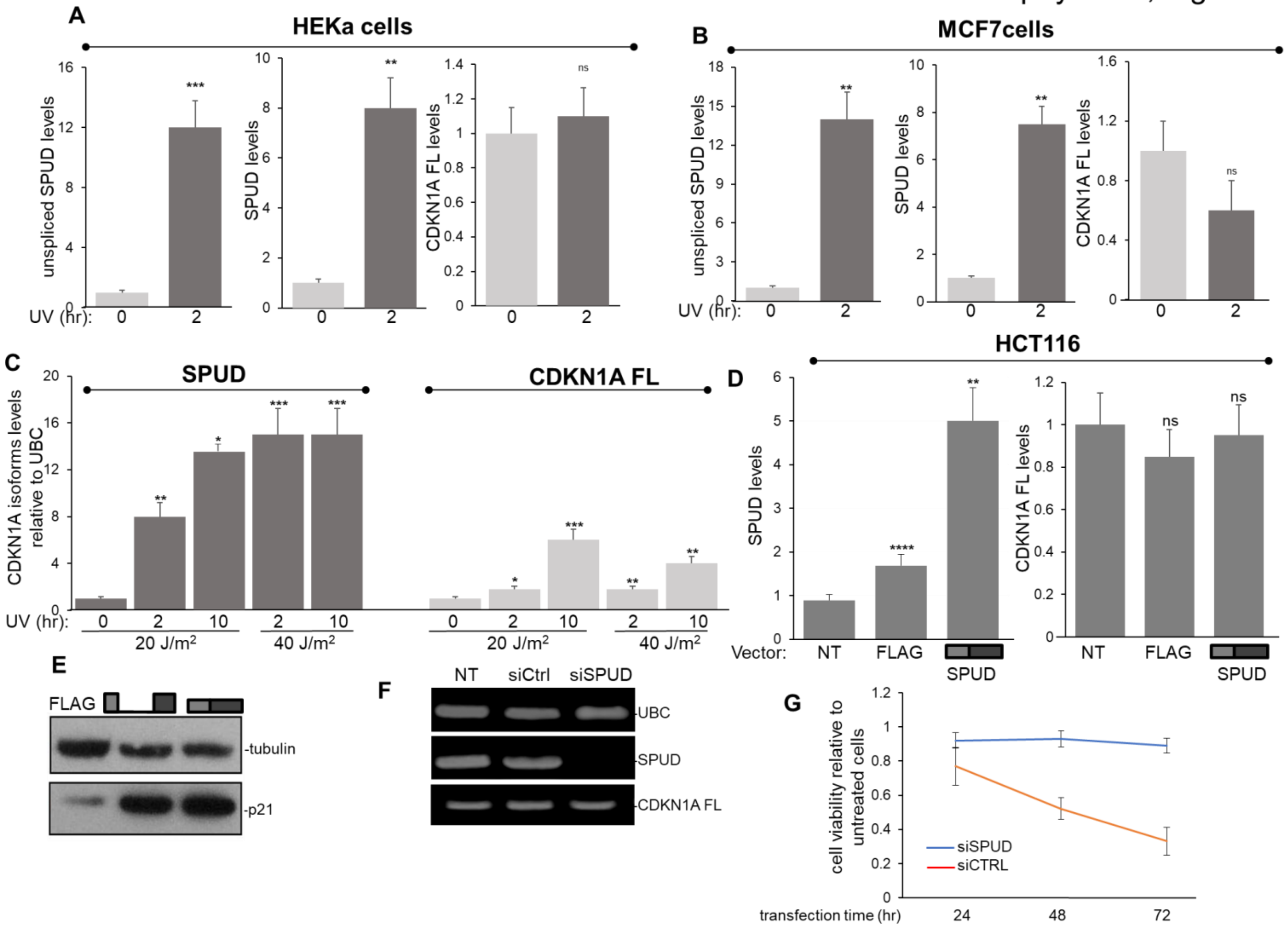

Supplemental Figure 3. (A-B) Effect of UV treatment on the levels of “unspliced SPUD”, SPUD and CDKN1A “unspliced SPUD”, SPUD and full-length CDKN1A in HEKa keratinocytes (A) and MCF7 breast cancer cells. UV induces APA without inducing full-length in MCF7 breast cancer cells. Cultured cells were exposed to 20J/m^2 UV and allowed to recover for 2 h. RNA was extracted and qRT-PCR analysis performed for the indicated isoforms (n=3). **(C)** SPUD levels depend on intensity of UV treatment. The effect on *CDKN1A* isoforms levels in HCT116 cells of two UV-C doses (20 J/m^2 and 40 J/m^2) after 2 and 10 h recovery time after the treatment was analyzed. An increase in UV-C intensity resulted in higher levels of SPUD and CDKN1A full-length at both 2 and 10 h recovery time relative to non-treated cells. The increase for both SPUD and CDKN1A with UV-C 20 J/m^2 are similar to those shown in Figure 2A. **(D)** SPUD overexpression leads to higher SPUD without affecting full-length mRNA levels. HCT116 cells were transfected with mammalian expression vector containing SPUD cDNA. After 48 h, cells were lysed and total RNA was extracted for analysis for CDKN1A isoforms. SPUD levels after exposure to UV treatment of different intensity in the previous submission. In the new Supplementary Figure S3 (panel D), we analyzed the effect of two UV-C doses (20 J/m^2 and 40 J/m^2) on SPUD and CDKN1A full-length levels in HCT116 cells after 2 and 10 h recovery time after the treatment. An increase in UV-C intensity resulted in higher levels of SPUD and CDKN1A full-length at both 2 and 10 h recovery time relative to non-treated cells. The increase for both SPUD and CDKN1A with UV-C 20 J/m^2 are similar to those shown in Figure 2A. **(E)** Overexpression of both unspliced SPUD and SPUD lead to upregulation of p21 protein levels. HCT116 cells were transfected with either empty FLAG-containing vector one of unspliced and spliced SPUD. Whole cell extracts were prepared and resolved by SDS-PAGE before probing for p21 or tubulin loading control (n=3). ns, not significant; ** *$P<0.001$; **** $P<0.00001$.* **(F)** siSPUD can deplete SPUD expression without significantly affecting CDKN1A full-length expression. Cytoplasmic RNA from HCT116 cells treated with siSPUD was analyzed by 5’ RACE using a primer located in the 5’ UTR of the CDKN1A gene. 5’ RACE results were similar to the ones obtained with a 5’ forward primer in exon 1 for both CDKN1A full-length and SPUD lncRNA upon siRNA treatment. 5’ RACE products were separated on an agarose gel and detected by ethidium bromide staining. UBC amplification was used as loading control. Shown is a representative gel from three independent assays. **(G)** SPUD depletion diminished loss of viability caused by control siRNA transfection. Cells were transfected as in (C) and measured via trypan blue at the indicated timepoints post-transfection. A representative gel from three independent assays from three biological samples is shown (n=3).

Murphy et al., Figure S4

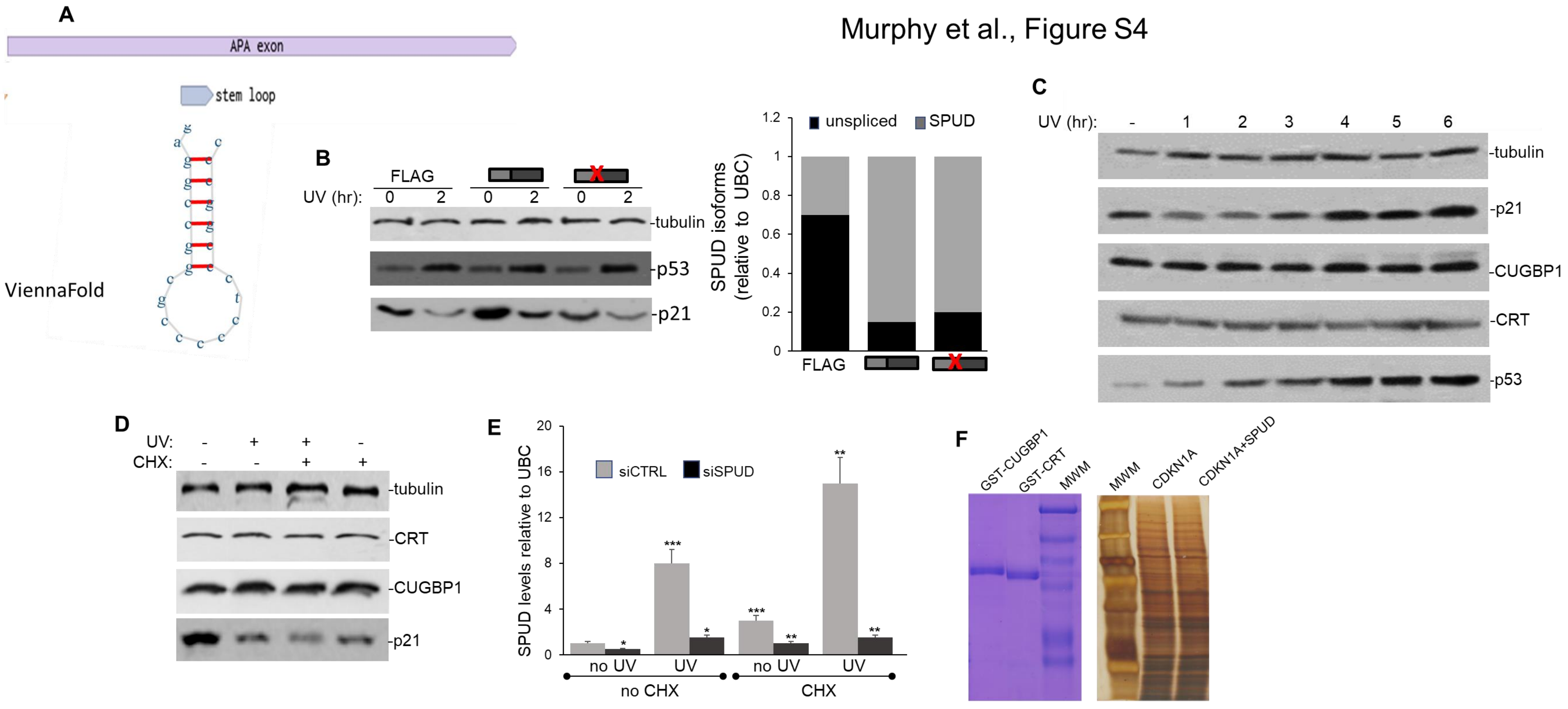

Supplemental Figure 4. (A) Potential CUGBP-binding stem loop within SPUD ALE. SPUD was analyzed using ViennaFold and stem-loop with the most consecutive base pairs was isolated. The ALE-loop is similar to the one previously described in exon 2 (73). The location of ALE stem loop within SPUD is shown. **(B)** A deletion of loop located in SPUD-ALE (ΔGGCCG derivative), which is not present in the full-length CDKN1A transcript, abolishes the SPUD-mediated induction of p21 protein levels. While the mRNA levels of the deletion mutant are similar to those of SPUD, no induction in p21 expression was observed with the deleted derivative. A representative gel from three independent assays from three biological samples is shown (n=3). **(C-D)** CUGBP and CRT levels are unchanged during UV-mediated DNA damage response (C) and cycloheximide (CHX) treatment (D). HCT116 cells were exposed to UV and allowed to recover for the described times prior to NE preparation. Samples were analyzed by Western blotting for the proteins indicated. A representative gel from three independent assays from three biological samples is shown (n=3). **(E)** UV and CHX treatment increase *SPUD* transcript levels with minimal change on full-length *CDKN1A* levels. HCT116 cells were depleted in SPUD as in Figure 6 and treated with either UV (20 J/m^2), CHX (2 μg/ml) or CHX (2 μg/ml)/UV (20 J/m^2) with 2 h recovery. qRT-qPCR was performed on RNA purified from cells treated in each condition. SPUD levels observed in each condition were normalized to the values of non-treated samples. Data shown is from 3 independent biological samples analyzed by triplicate, SD (n=3). *P<0.01; **P<0.001 and ***P<0.0001. **(F)** Coomassie Blue and Silver staining analysis of samples used in Figures 8E-F.

Murphy et al., Figure S5

Additional Primers	
Primer Name	Sequence (5' – 3')
U2 snRNA Forward	GGCCTTTTGGCTAAGATCAA
U2 snRNA Reverse	TATTCCATCTCCCTGCTCCA
p21 Exon 2 Forward	CTGGAGACTCTCAGGGTCGAAA
p21 Exon 3 Reverse	GATTAGGGCTTCCTCTTGGAGAA
HuR Forward	CAGAAGAGGCAATTACCAGTTTCAATGG
HuR Reverse	GCTTCTTCATAGTTTGTCATGGTCAC
GAPDH Forward	ACCACAGTCCATGCCATCAC
GAPDH Reverse	TCCACCACCCTGTTGCTGTA
CDKN1A Exon 1 Forward	ATGCGTGTTCGCGGGTGT
CDKN1A APA Exon Reverse	AGTGATGAGTCAGTTTCCTGCAAG
CDKN1A Intron 1 Reverse	AGGTGGTGGACACAGTGGCGTA
Ubiquitin C Forward	TGGCACAGCTAGTTCCGTCGCA
Ubiquitin C Reverse	CGAGGGTGATGGTCTTACCAGTC

Supplemental Figure 5. Panel of qRT-PCR oligonucleotides used in this study.
